# Organic Solar Cell With Efficiency Over 20% and *V*_OC_ Exceeding 2.1 V Enabled by Tandem With All‐Inorganic Perovskite and Thermal Annealing‐Free Process

**DOI:** 10.1002/advs.202200445

**Published:** 2022-07-20

**Authors:** Xiaoyu Gu, Xue Lai, Yuniu Zhang, Teng Wang, Wen Liang Tan, Christopher R. McNeill, Qian Liu, Prashant Sonar, Feng He, Wenhui Li, Chengwei Shan, Aung Ko Ko Kyaw

**Affiliations:** ^1^ Guangdong University Key Laboratory for Advanced Quantum Dot Displays and Lighting Department of Electrical & Electronic Engineering Southern University of Science and Technology Shenzhen 518055 P. R. China; ^2^ Department of Chemistry Southern University of Science and Technology Shenzhen 518055 P. R. China; ^3^ Department of Materials Science and Engineering Monash University Clayton Victoria 3800 Australia; ^4^ Center for Materials Science Queensland University of Technology Brisbane Queensland 4000 Australia

**Keywords:** electron transporting layers, energy loss, inter‐connecting layers, perovskites/organic tandem solar cells

## Abstract

Organic solar cells (OSCs) based on polymer donor and non‐fullerene acceptor achieve power conversion efficiency (PCE) more than 19% but their poor absorption below 550 nm restricts the harvesting of high‐energy photons. In contrast, wide bandgap all‐inorganic perovskites limit the absorption of low‐energy photons and cause serious below bandgap loss. Therefore, a 2‐terminal (2T) monolithic perovskite/organic tandem solar cell (TSC) incorporating wide bandgap CsPbI_2_Br is demonstrated as front cell absorber and organic PM6:Y6 blend as rear cell absorber, to extend the absorption of OSCs into high‐energy photon region. The perovskite sub‐cell, featuring a sol–gel prepared ZnO/SnO_2_ bilayer electron transporting layer, renders a high open‐circuit voltage (*V*_OC_). The *V*_OC_ is further enhanced by employing thermal annealing (TA)‐free process in the fabrication of rear sub‐cell, demonstrating a record high *V*_OC_ of 2.116 V. The TA‐free Ag/PFN‐Br interface in organic sub‐cell facilitates charge transport and restrains nonradiative recombination. Consequently, a remarkable PCE of 20.6% is achieved in monolithic 2T‐TSCs configuration, which is higher than that of both reported single junction and tandem OSCs, demonstrating that tandem with wide bandgap all‐inorganic perovskite is a promising strategy to improve the efficiency of OSCs.

## Graphical abstract

Thermalization loss limits the efficiency of OSCs. By integrating with wide bandgap CsPbI_2_Br, a tandem solar cell is demonstrated with a remarkable voltage of 2.116 V with ≈0.001 V voltage loss through a TA‐free process in depositing of organic rear cell. This work demonstrates the great potential of tandem solar cell incorporating perovskite and organic absorber.

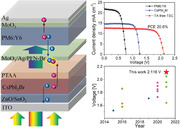

## 1 Introduction

Organic solar cells (OSCs) have made great progress during the last few years along with the emergence of small molecular non‐fullerene acceptors (NFAs).^[^ ^1^ ^]^ With continual efforts in materials design, device engineering, and photophysics study, the power conversion efficiency (PCE) has already exceeded 19%.^[^ ^1^, ^2^ ^]^ However, most organic materials have relatively narrow optical absorption with poor photoresponse in either near‐infrared (NIR) or near‐ultraviolet (NUV) region. To extend the light absorption, various low bandgap (LBG) NFAs together with the multicomponent (ternary/quaternary) strategy have been proposed and achieved impressive results with high short‐circuit current (*J*_SC_),^[^ ^3^ ^]^ but the intrinsic energy loss derived from the hot exciton relaxation of high energy photons cannot be minimized in single junction OSCs.^[^ ^4^ ^]^ In addition, NFAs barely absorb NUV light, in contrast to fullerene acceptors, while corresponding high‐performance donors also have very poor absorption below 500 nm, for instance, well‐known PM6 and PBDB‐T.^[^ ^5^ ^]^ Looking at cross‐disciplinary photovoltaic technology, solar cells based on organic–inorganic halide perovskites, which render higher efficiency than OSCs, exhibit much stronger absorption below 500 nm.^[^ ^6^ ^]^ This clearly reveals that high energy photons are not fully utilized in the start‐of‐the‐art OSCs, which limits the further improvement of their efficiency.

Tandem solar cells (TSCs) composed of wide bandgap (WBG) and LBG semiconductors in series have a broad absorption spectrum with each semiconductor in sub‐cell responsible for separate absorption regions; and hence, the thermalization loss and below bandgap loss are reduced, leading to an improved photovoltaic performance.^[^ ^7^ ^]^ The two most widely studied architectures of tandem device are two‐terminal (2T) and four‐terminal (4T). The mechanically stacked 4T tandem solar cells can be easily fabricated due to the electrically independent sub‐cells, which provides flexibility in absorber choice. However, the additional semitransparent contact, normally sputtered transparent conducting oxides, causes the undesired reflection and parasitic absorption of incident light which lowers the PCE, not to mention the additional manufacturing cost of dichroic mirror in 4T spectral splitting tandem device.^[^ ^7a^ ^]^ In 2T architecture, sub‐cells are electrically connected in series with one semitransparent interconnecting layer, resulting in lower parasitic absorption. The monolithic integration of 2T‐ TSCs allows sequential layer‐by‐layer film deposition and therefore, an easy module integration. In monolithic 2T‐TSCs, the photocurrent is limited by the minimum value of sub‐cells while the open‐circuit voltage should be equal to the sum of the voltages of sub‐cells.^[^ ^8^ ^]^ Therefore, it is critical to balance the current by achieving complementary absorption and at the same time, reduce energy loss to acquire maximum voltage. However, the development of high‐performance WBG organic materials seriously lags behind the LBG semiconductors, with the existing WBG organic solar cells suffering high energy loss (*E*_loss_ = *E*_g_ − *qV*_oc_, where *E*_g_ is the bandgap, *q* is the elementary charge, and *V*_OC_ is the open circuit voltage) and poor photoresponse below 550 nm.^[^ ^9^ ^]^ All‐inorganic perovskites (CsPb*X*_3_, *X* = Cl, Br, I) could be considered as promising candidates for front sub‐cell to complement organic cells in tandem structure due to their adjustable wide bandgap (1.7–2.3 eV) and excellent thermal tolerance.^[^ ^10^ ^]^ High quality all‐inorganic perovskites possess high photoresponse with maximum EQE reaching 90% within ≈300–500 nm, which ensures the current of front cell breaks the bottleneck.^[^ ^11^ ^]^ The cubic *α*‐CsPbI_3_ with a direct bandgap of ≈1.7 eV has demonstrated the highest PCE of 20.37% but the non‐ideal Goldschmidt tolerance factor of ≈0.8 leads to a spontaneous phase transition from *α* to non‐perovskite *δ* phase.^[^ ^11b^ ^]^ By doping with Br, the Br/I mixed halide CsPbI_2_Br merits excellent photovoltaic performance as well as thermally stable phase at room temperature. The highest reported PCE for CsPbI_2_Br‐based solar cell has exceeded 17%,^[^ ^12^ ^]^ with the highest voltage reaching 1.43 V,^[^ ^13^ ^]^ approaching the predicted *V*_OC_ (1.62 V) according to Shockley–Queisser (SQ) limit.^[^ ^14^ ^]^ Besides, different from other inorganic photovoltaic semiconductors with high‐energy intensive processing and emission of toxic by‐products such as CdTe, CIGS, GaAs, and Si,^[^ ^15^ ^]^ CsPbI_2_Br features a simple solution‐processed technique with feasible thermal treatment temperature of wide range (≈100–300 °C).^[^ ^16^ ^]^ Given a wide bandgap of 1.92 eV, CsPbI_2_Br demonstrates a great potential for application in semitransparent and tandem solar cells. Ding, et al. first applied CsPbI_2_Br as front absorber to collect high‐energy photons for PTB7‐Th:CO*_i_* 8DFIC:PC_71_BM ternary blend, and the 2T‐TSC demonstrated a PCE of 15.04%.^[^ ^17^ ^]^ Efficiency was further improved to 18.4% by Yip and co‐workers by introducing PM6:Y6 as light absorber in organic rear cell.^[^ ^18^ ^]^ Recently, Wang et al. and Li et al. have subsequently pushed the efficiency boundary over 21% by applying polyTPD in interconnecting layer (ICL) and by surface reconstruction of perovskite using trimethylammonium chloride, respectively, realizing efficient charge extraction and recombination in ICL.^[^ ^19^ ^]^ Although considerable efficiency has been achieved, the voltage loss within ICL remains high and could be further reduced.

Here, in this work, we applied all‐inorganic perovskite CsPbI_2_Br as a light absorber of front sub‐cell, in which the relatively wide bandgap of 1.92 eV could effectively absorb photons in the NUV and visible region and leave the lower energy photon to be absorbed by the rear cell. By inserting a sol–gel prepared ZnO layer (s‐ZnO) together with SnO_2_ as a bilayer electron transporting material, the single junction perovskite solar cell obtained an efficient electron extraction and a relatively high *V*_OC_ which could improve the overall tandem cell performance. The narrow bandgap PM6:Y6 bulk‐heterojunction (BHJ) film was introduced as a rear cell absorber to complement with WBG CsPbI_2_Br and extend the absorption beyond 900 nm. Different from common approach in single junction BHJ solar cell, the device performance of rear sub‐cell is improved by thermal annealing (TA)‐free approach. By systematically investigating the TA effect on the organic rear cell, we discovered that TA‐free process reduces the contact resistance due to the efficient charge transport at the electrode/PFN‐Br interface; and hence, suppresses the charge accumulation and nonradiative recombination. Therefore, TA‐free device enhances *V*_OC_ by hundreds of millivolts without compromising the *J*_SC_ and FF. Moreover, TA‐free process increases the conductivity of ICL. By employing MoO_3_/Ag/PFN‐Br as an ICL, a monolithic 2T‐TSC yields a PCE of 20.6% with the highest *V*_OC_ reaching 2.116 V, which is approximately the submisson of the V_OC_ of individual subcells with only 0.001V difference due to the suppressed nonradiative recombination at the interfaces. This is the highest *V*_OC_ ever reported based on perovskite/organic absorber, showing a bright prospect for such device configuration. Moreover, the result also surpasses the highest reported PCEs of both single junction and tandem organic solar cells,^[^ ^1^, ^20^ ^]^ demonstrating that tandem with WBG all‐inorganic perovskite is an effective and innovative strategy for utilizing broad solar spectrum from NUV to NIR, in turn, improving the efficiency of OSCs.

## 2 Results and Discussion

**Figure 1:**
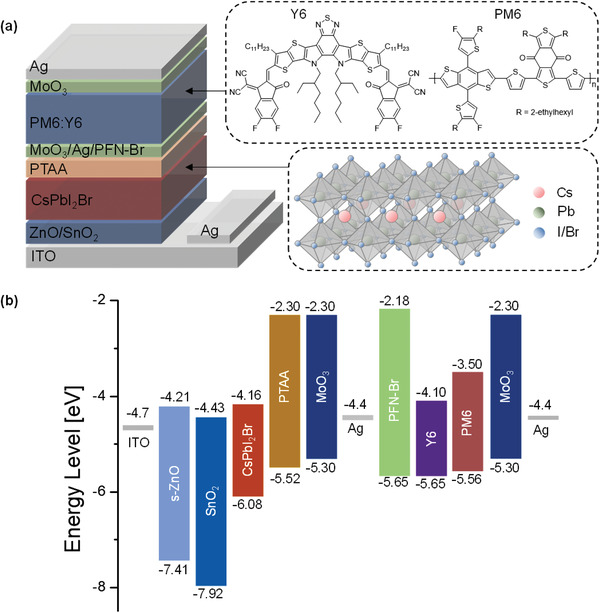
a) Device structure and b) corresponding energy levels of individual layers in 2T inorganic perovskite/organic TSCs.

**Figure 2:**
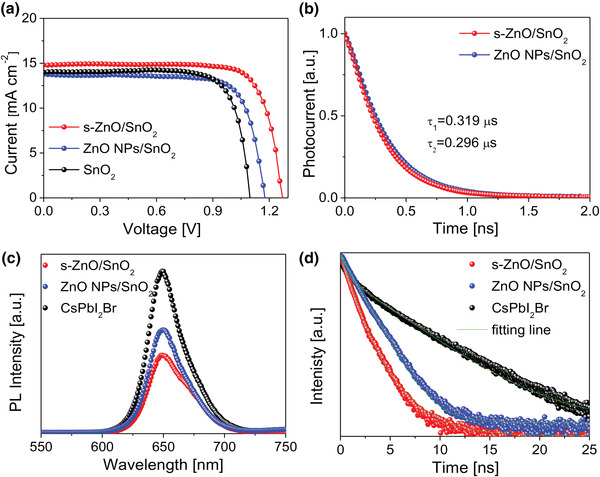
a) *J–V* characteristics of all‐inorganic perovskite front sub‐cells with different ETLs. b) Transient photocurrent measurement of the devices based on s‐ZnO/SnO_2_ and ZnO NPs/SnO_2_ ETLs. c) Photoluminescence spectra and d) time‐resolved photoluminescence decay curves of bare CsPbI_2_Br film and that deposited on s‐ZnO/SnO_2_ and ZnO NPs/SnO_2_ ETLs.

We fabricated the 2T‐perovskite/organic TSCs with a structure of ITO/ZnO/SnO_2_/CsPbI_2_Br/MoO_3_/Ag/PFN‐Br/PM6:Y6/MoO_3_/Ag. The schematic diagram of tandem device structure and energy levels of individual layers used in the device are shown in **Figure** **1**. The energy levels of respective layers are taken from previous reports.^[^ ^21^ ^]^ To obtain high‐efficiency perovskite/organic TSCs, we needed to maximize the voltage of individual sub‐cells as well as balance the current in the sub‐cells due to the series connection of two sub‐cells in the 2T‐TSCs. Therefore, we optimized the performance of individual sub‐cells separately before constructing tandem cell. First, we started with front cell of all‐inorganic perovskite solar cell. Various bilayer combinations based on ZnO were reported as electron transport layer to minimize energy loss (*E*_loss_) by reducing surface work function, creating interfacial dipoles, improving perovskite crystallinity, and so on.^[^ ^22^ ^]^ Therefore, in this work, we inserted a sol–gel prepared ZnO (s‐ZnO) layer beneath the commonly used SnO_2_ layer to reduce the interfacial *E*_loss_. ZnO nanoparticles (ZnO NPs) were also applied as comparison according to previous reports.^[^ ^18^, ^21^ ^]^ The front sub‐cell was fabricated with a structure of ITO/ZnO/SnO_2_/CsPbI_2_Br/PTAA/MoO_3_/Ag, and the energy level of individual layers can be referred to in Figure 1a. The CsPbI_2_Br film was formed by a two‐step sequential annealing process. The absorption spectra of perovskite films and the corresponding Tauc plots (Figure S1a,b, Supporting Information) indicate that the CsPbI_2_Br films deposited on different electron transporting layers (ETLs) (SnO_2_, ZnO NPs/SnO_2_, and s‐ZnO/SnO_2_) possess the same bandgap of 1.92 eV, suggesting the identical semiconducting properties. The current density–voltage (*J*–*V*) curves of the best performing devices are presented in **Figure** **2**a, and the detailed data are summarized in Table S1, Supporting Information. SnO_2_‐based devices generated a PCE of 12%, with a *J*_SC_ of 14.00 mA cm^−2^, *V*_OC_ of 1.097 V, and FF of 77.8%. ZnO NPs/SnO_2_ bilayer improves the *V*_OC_ to 1.177 V while s‐ZnO/SnO_2_ bilayer can simultaneously increase *J*_SC_, *V*_OC_, FF, and PCE to 14.79 mA cm^−2^, 1.271 V, 78.1% and 14.7%, respectively. The enhanced *V*_OC_ in s‐ZnO‐based devices implies that nonradiative recombination is suppressed.^[^ ^23^ ^]^ To understand different device performances of ZnO NP‐ and s‐ZnO‐based devices, scanning electron microscopy (SEM) was carried out. Comparing the top view of ZnO NPs/SnO_2_ with s‐ZnO/SnO_2_ (Figure S2a,b, Supporting Information), the ZnO NPs/SnO_2_ film exhibits a porous‐like morphology with noticeable pinholes, which could potentially lead to undesirable contact between electrode and the penetrated perovskite, in turn, causing leakage current and recombination at the interface, while s‐ZnO/SnO_2_ is much denser and can provide an effective hole blocking layer. Figure S2c,d, Supporting Information, shows the perovskite films deposited on the corresponding ETLs. It can be noticed that the surface of perovskite grown on ZnO NPs/SnO_2_ (Figure S2c, Supporting Information) is covered with cracks, which will bring energetic disorder and hinder charge transportation and thus, cause *E*_loss_ under illumination.^[^ ^24^ ^]^ Cracks are also responsible for moisture penetration and film decomposition.^[^ ^25^ ^]^ On the contrary, the s‐ZnO/SnO_2_ based film is much more uniform with negligible crack pattern, showing a better crystallization process. To evaluate the charge extraction and recombination dynamics of ZnO NPs/SnO_2_ and s‐ZnO/SnO_2_ bilayered ETLs, transient photocurrent (TPC) measurements, steady‐state photoluminescence (PL), and time‐resolved photoluminescence decay (TRPL) were conducted. TPC measurement (Figure 2b) reveals that the s‐ZnO/SnO_2_ based device exhibits a faster electron extraction time (0.296 µs) than that of ZnO NPs/SnO_2_‐based device (0.319 µs). The efficient charge extraction in s‐ZnO/SnO_2_‐based device was further confirmed by PL measurement of perovskite films deposited on various ETLs to rule out the influence of hole transporting layers, as shown in Figure 2c. The perovskite on s‐ZnO/SnO_2_ bilayer ETL exhibits a higher PL quenching than the perovskite on ZnO NPs/SnO_2_ ETL does. The corresponding carrier lifetime was calculated by a second‐order exponential fitting of the TRPL spectrum in Figure 2d. The lifetimes of sole perovskite film, perovskite on ZnO NPs/SnO_2_, and perovskite on s‐ZnO/SnO_2_ are 2.78, 1.73, and 1.11 ns, respectively, clearly showing that the excited electrons are extracted instantly at the ETL/CsPbI_2_Br interface and charge extraction is more efficient at s‐ZnO/SnO_2_ and CsPbI_2_Br interface. From the above results, s‐ZnO is considered as a more suitable material than ZnO NPs to combine with SnO_2_ as ETL. Besides, relatively small size of ZnO NPs usually have a strong absorption due to higher particle concentration and surface states density.^[^ ^26^ ^]^ As shown in Figure S3a, Supporting Information, the stronger absorption of ZnO NPs would cause undesirable absorption competition between ETL and perovskite layer in a regular n‐i‐p device structure and therefore, lower the external quantum efficiency (Figure S3b, Supporting Information). Due to the higher photovoltaic parameters of s‐ZnO/SnO_2_‐based device in single junction cell, we selected s‐ZnO/SnO_2_ bilayer as ETL of front cell in tandem cell.

**Figure 3:**
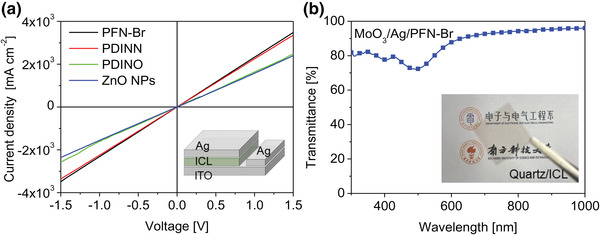
a) *J–V* curves of various ICL layers; MoO_3_/Ag/PFN‐Br, MoO_3_/Ag/PDINN, MoO_3_/Ag/PDINO, and MoO_3_/Ag/ZnO NPs. Inset shows the structure used for the test. b) Transmittance spectrum of MoO_3_/Ag/PFN‐Br ICL.

To realize a complementary absorption between sub‐cells, we chose PM6:Y6 blend as rear cell absorber; the absorption spectra of individual sub‐cells are depicted in Figure S4, Supporting Information. The relatively small bandgap of PM6:Y6 blend extends the light harvesting range toward 900 nm. First, we prepared single junction OSC with structure of ITO/PEDOT:PSS/PM6:Y6/MoO_3_/Ag to evaluate the photovoltaic performance of PM6:Y6. The *J*–*V* curves are presented in Figure S5a, Supporting Information; the single junction OSC exhibits a PCE of 15.64% with a *V*_OC_ of 0.845 V, *J*_SC_ of 25.57 mA cm^−2^, and FF of 72.41%, which is comparable to the reported devices based on PM6:Y6.^[^ ^27^ ^]^ The EQE spectrum (Figure S5b, Supporting Information) reveals that PM6:Y6 blend has a considerable photovoltaic response with cutoff ≈950 nm, showing a well‐matched absorption with CsPbI_2_Br. Subsequently, we applied MoO_3_/Ag/PFN‐Br as interconnecting layer (ICL) for tandem cell fabrication because such combination renders the highest vertical conductivity among the various interfacial materials to connect two sub‐cells with low resistance (**Figure** **3**a). In 2T‐TSCs, sub–cells are electrically connected by ICL, also known as the recombination layer. Photogenerated carriers in absorbers are separated between a sandwich designed selective contacts and extracted by the corresponding electrodes, leaving the ICL collecting the holes from one sub‐cell and electrons from the other sub‐cell. Therefore, a high conductivity ICL is essential for efficient recombination. The PFN‐Br based ICL has a transmittance over 90% beyond 650 nm that guarantees the light absorption of organic sub‐cell (Figure 3b). Additionally, we studied the effect of TA on the conductivity of ICL because TA is usually necessary to improve the crystallinity and phase separation of BHJ film in the rear cell and to improve the charge transport. As shown in Figure S6, Supporting Information, after annealing at 110 °C for 10 min, the conductivity drastically declines, which might adversely affect the charge collection efficiency and carrier recombination at ICL. The variation in conductivity could be attributed to the thermal vulnerability of Ag/PFN‐Br interface, which was confirmed later in the series of experiments.

**Figure 4:**
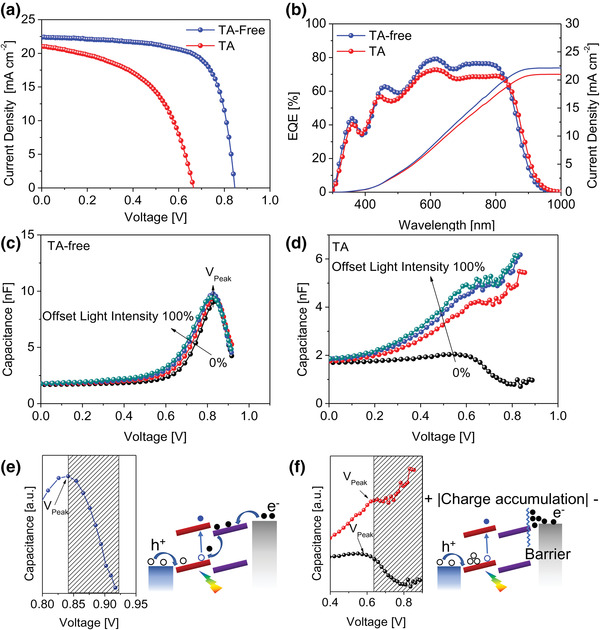
a) *J–V* curves and b) corresponding EQE spectrum of TA and TA‐free organic rear sub‐cells based on ITO/Ag/PFN‐Br substrates. Capacitance–voltage measurements with light illumination intensity from 0–100% and schematic illustration of charge injection process at the applied voltages exceeding *V*_peak_ for c,e) TA‐free and d,f) TA devices.

Furthermore, it is critical to optimize the performance of OSC in the same device structure as rear cell in tandem cell. Therefore, next we prepared the OSC with device structure of ITO/PFN‐Br/PM6:Y6/MoO_3_/Ag to optimize the performance of rear sub‐cell. The *J*–*V* characteristics of devices in Figure S7, Supporting Information, show S‐shaped curves with kinks near the open circuit region, regardless of processing conditions. It can be attributed to the inefficient charge transport between ITO substrate and PFN‐Br layer, causing charge accumulation and thus, creating space charge region at the interface, which is also observed in previous papers.^[^ ^28^ ^]^ We found that insertion of a thin Ag layer (1 nm) can form ohmic contact and eliminate the kink in the *J–V* curve. As shown in Figure S8, Supporting Information, UPS measurement verifies the work function (WF) shift from −4.4 eV for Ag to −4.12 eV for PFN‐Br/Ag due to interfacial dipole pointing from PFN‐Br to Ag. A small energy gap of 50 meV between WF of Ag and HOMO of Y6 facilitates charge extraction and at the same time, minimizes the energy loss. Based on this, we fabricated device on ITO/Ag/PFN‐Br substrate to optimize the performance of rear organic sub‐cell. Generally, thermal annealing process is necessary to improve the performance of organic BHJ solar cell. Nevertheless, to our surprise, different from common phenomenon in single junction BHJ device, we realized that TA‐free process improves the efficiency of rear organic sub‐cell significantly. TA‐free device exhibits a PCE of 13.4% with *J*_SC_ of 22.37 mA cm^−2^, *V*_OC_ of 0.846 V, and FF of 70.7% while TA device shows a PCE of 7.2% only with *J*_SC_ of 21.04 mA cm^−2^, *V*_OC_ of 0.665 V, and FF of 51.7% (**Figure** **4**a; Table S2, Supporting Information). The enhanced EQE spectrum of TA‐free device agrees well with *J*_SC_ increase (Figure 4b).

In addition, the substantial increase in FF of TA‐free device is also consistent with double decrease in series resistance (*R*_S_) and triple increase in shunt resistance (*R*_SH_), compared with TA device. The decrease in *R*_S_ further implies the reduced contact resistance due to efficient charge transfer at interface in TA‐free device. To assess the charge transfer characteristics, we conducted capacitance–voltage (*C–V*) and electrochemical impedance spectrum (EIS) measurements. *C–V* characteristics usually reveal the surface charge accumulation of photogenerated carriers due to potential barrier under illumination.^[^ ^29^ ^]^ As shown in Figure 4c, in TA‐free device, upon varying the light illumination intensities from ≈0–100%, the constant capacitance under low bias voltage is observed due to the geometric capacitance with the value ≈1.7 nF. Further increase in applied bias (*V*_ap_) leads to a decrease in built‐in electric field, which lessens the driving force for charge carriers moving toward the corresponding electrodes and causes charge accumulation at the interface. This behavior can be seen as a sharp increase in capacitance in the *C–V* curve. Continued increase in *V*_ap_ until surpassing the built‐in potential (*V*_peak_ = 0.841 V) leads to charge injection. Figure 4e,f illustrates different charge injection mechanisms in the shaded regions of *C–V* curves in the TA and TA‐free devices. In the case of TA‐free device (Figure 4e), the injected charges start to recombine with the photogenerated carriers, resulting in the drop of capacitance. However, in the case of TA device (Figure 4f), the capacitance values keep uptrend along with increasing applied voltage, suggesting that the presence of barrier at the interface prevents the injection of electrons into photoactive layers.

**Figure 5:**
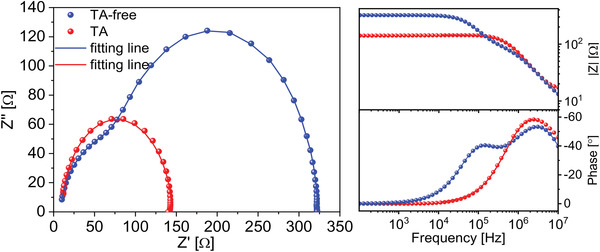
EIS measurement for TA and TA‐free organic rear sub‐cells depicted as Nyquist plot (left) and Bode plot (right).

EIS measurement was conducted to further explore the interfacial charge transport in TA and TA‐free devices. The Cole–Cole Nyquist plot and corresponding Bode plot measured with frequencies ranging from 10 MHz to 1 Hz at bias of *V*_OC_ are shown in **Figure** **5**. Two charge transport processes can be identified in the TA‐free device, represented by two semi‐circles in the Nyquist plot. The processes can be recognized by the characteristic peaks in the Bode plot (Figure 5, bottom right), in which a high frequency peak at ≈2.5 × 10^6^ Hz and a low frequency peak at ≈1.5 × 10^5^ Hz are observed in TA‐free device. However, only one peak at high frequency is identified after TA, indicating only one charge transport process. The missing semi‐circle can be attributed to the inefficient charge transfer at degraded interfacial layer or lack of proper interfacial layer according to previous reports.^[^ ^30^ ^]^ We also compared the impedance curve for TA and TA‐free devices based on ITO/PFN‐Br and ITO/Ag/PFN‐Br substrates. Both semi‐circles can be clearly seen before and after TA in ITO/PFN‐Br devices (Figure S9, Supporting Information), and therefore, the missing semi‐circle located at low frequency was confirmed originating from the ITO(Ag)/PFN‐Br interface. Considering the above observation, we fitted the EIS data with equivalent circuit shown in Figure S9c, Supporting Information, and the fitted parameters are presented in Table S3, Supporting Information. The fitted curves showed a reasonable concordance with original spectrum. *R*_s_' accounts for ohmic losses at contacts, which is in series with a charge transport resistance (*R*_1_) and capacitor CPE_1_ in parallel. CPE stands for constant phase element which describes non‐ideal capacitors, and contains pseudo capacitance (CPE_T_) and corresponding quality factor (CPE_P_). For TA‐free device, with the presence of additional charge transport process, *R*_2_ and CPE_2_ are required to provide accurate fitting of the spectra.^[^ ^30^ ^]^ TA‐free device exhibits a lower *R*_S’_ (8.52 Ω), indicating smaller contact resistance at interface that reduces voltage loss caused by non‐ohmic contact, which is consistent with the smaller radius of the semi‐circle at high frequency in Nyquist plot. Moreover, *R*_2_, which refers to the charge transport resistance, is significantly increased from 68.9 to 135.5 Ω after annealing, implying the rise of interfacial resistance during annealing, which is consistent with the FF and *J*_SC_ performance for TA devices.

**Figure 6:**
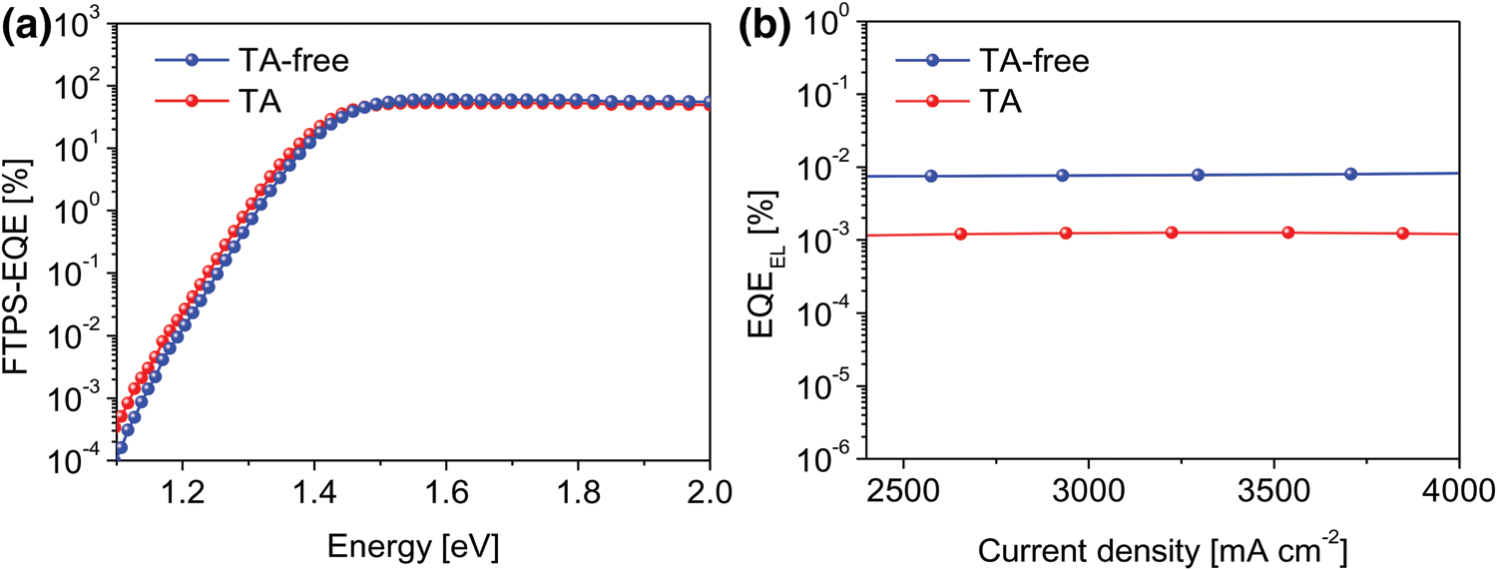
a) FTPS–EQE and b) EQE_EL_ for TA and TA‐free organic rear sub‐cells.

Inefficient charge transport causes undesired charge recombination; therefore, the energy losses for TA and TA‐free devices were systematically studied. Fourier transform photocurrent spectroscopy (FTPS‐EQE) and electroluminescence (EQE_EL_) were performed (**Figure** **6**) and the various energy losses (Δ*E*_1_, Δ*E*_2_ and Δ*E*_3_) in the photovoltaic system were calculated (Figure S10 and Table S4, Supporting Information). The bandgap $E_{g}^{\mathrm{PV}}$, determined from EQE spectrum in Figure 3b, are 1.47 and 1.49 eV for TA and TA‐free PM6:Y6 BHJ films, respectively. $\Delta E_{1}\left(E_{g}-eV_{\mathrm{OC}}^{\mathrm{SQ}}\right)$, represents the radiative recombination originating from above bandgap absorption,^[^ ^31^ ^]^ displays almost identical value for TA (0.262 eV) and TA‐free (0.261 eV) devices. $\Delta E_{2}\left(eV_{\mathrm{OC}}^{\mathrm{SQ}}-eV_{\mathrm{OC}}^{\mathrm{rad}}\;\right)$, caused by the below bandgap absorption, is derived from FTPS‐EQE (Figure 6a); the calculated values are also quite close for TA (0.093 eV) and TA‐free (0.091 eV) devices. Most importantly, the nonradiative recombination calculated from the equation Δ*E*_3_ = −*kT*ln(EQE_EL_), is critical to the voltage output. From the experimental measurement (Figure 5b), the EQE_EL_ values for TA and TA‐free devices are determined to be≈1.24 × 10^−4^ and ≈7.66 × 10^−4^, respectively. The extracted Δ*E*_3_ for TA‐free device is 0.245 eV, which is much lower than 0.292 eV for TA device. The suppressed nonradiative recombination is in accordance with the lower interfacial resistance of TA‐free device, resulting in tremendous increase in output voltage.

To reveal the possible physical changes at the interface upon TA, we studied X‐ray photoelectron spectrum (XPS) of Ag on the ITO/Ag/PFN‐Br substrates. As is shown in Figure S11, Supporting Information, a noticeable shift toward lower binding energy is observed in TA‐treated ITO/Ag/PFN‐Br, compared with TA‐free ITO/Ag and ITO/Ag/PFN‐Br substrates, suggesting the reaction of Ag and generated Ag ions with higher oxidation state during annealing.^[^ ^32^ ^]^ However, due to the very low content of such Ag ions, it is difficult to determine the specific substance.

Considering the effect of annealing on the crystallinity and morphology of the BHJ film, we conducted atomic force microscopy (AFM) and grazing‐incidence wide‐angle X‐ray scattering (GIWAXS) measurements. The root‐mean‐square (RMS) roughness of TA‐free film is 1.69 nm for the scan area of 2 µm × 2 µm, whereas that of TA film increases to 1.98 nm after annealing at 110 °C for 10 min (Figure S12, Supporting Information). The RMS differences can be ascribed to the difference ratios of PM6 to Y6 on the surface of the two films caused by phase separation along the vertical direction during annealing.^[^ ^33^ ^]^ As the neat Y6 film is several times rougher than PM6 film,^[^ ^33^ ^]^ the lower RMS top surface of the TA‐free film suggests the vertical phase separation with a PM6‐rich component at the upper film, which is conducive to the hole extraction from the PM6:Y6 blend film to top MoO_3_/Ag electrode.^[^ ^33^, ^34^ ^]^ GIWAXS was conducted to further characterize the molecular packing of the blend film. The 2D GIWAXS patterns and the corresponding scattering intensity data (Figure S12c–e, Supporting Information) reveals that the TA‐PM6:Y6 blend film has a stronger scattering peak located at *q*_z_ = 1.77 Å (*d* = 3.55 Å) along the out‐of‐plane (OOP) direction, suggesting the preferential face‐on orientation and enhanced *π*–*π* stacking of Y6 after annealing.^[^ ^1a^ ^]^ Hence, a red shift in absorption peak of Y6 from 802 nm for TA‐free film to 814 nm for TA film can be seen (Figure S13, Supporting Information); the larger bandgap of TA‐free film is also one of the reasons for the voltage increase. Besides, in the in‐plane (IP) direction, an enhanced scattering peak can be found at *q_xy_* = 0.298 (*d* = 21.08 Å) and *q_xy_* = 0.432 (*d* = 14.54 Å) in the TA film compared with TA‐free film, confirming the enhanced molecular stacking along the edge‐on orientation, which however, will not contribute to the overall charge collection in the vertical‐transport device such as solar cell. Therefore, the overall increase in crystallinity does not compensate the energy loss at the interface.

**Figure 7:**
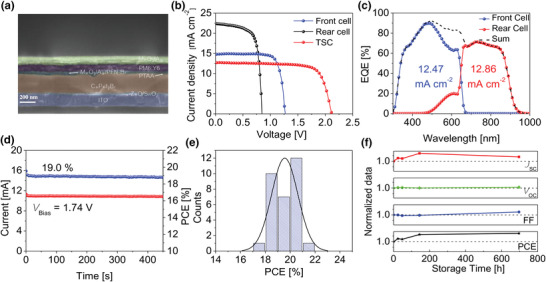
a) Cross‐section SEM image of TSC. b) *J–V* curves of optimal individual sub‐cells and TA‐free TSC. c) EQE spectra and d) MPP tracking of TA‐free TSC measured in glovebox. e) PCE distribution histogram of 31 devices from the same batch; detailed data is listed in Table S2, Supporting Information. f) the variation in *J*_SC_, *V*_OC_, FF, and PCE of TA‐free TSC over storage time during stability test.

Finally, we fabricated the 2T‐perovskite/organic TSCs. The detailed fabrication process can be referred to in the experimental section and Figure S14, Supporting Information. The thickness of each layer was extracted from the cross‐section SEM image (**Figure** **7**a). The thicknesses of CsPbI_2_Br and PM6:Y6 layers were controlled to be ≈300 nm and ≈100 nm, respectively, to match the current density of sub‐cells. Each layer in the SEM image is distinctly recognizable, showing that the proper solvent usage allows each layer to be deposited well without damaging the underlying layer. We first compared the photovoltaic performance of tandem cells based on TA and TA‐free organic sub‐cells, and their *J*–*V* curves are presented in Figure S15a, Supporting Information. It can be clearly seen that the TA‐free device outperforms the TA device in every parameter, especially *V*_OC_, which can be attributed to the higher *V*_OC_ in TA‐free organic sub‐cell. The TA‐free device also exhibits a lower dark current density of 3.1 × 10−5 mA cm^−2^ compared with 8.2 × 10−5 mA cm^−2^ for TA device (Figure S15b), confirming the higher voltage of TA‐free device according to the following equation:^[^ ^35^ ^]^

(1) $$V_{\mathrm{OC}}=\frac{k_{B}T}{q}\ln\left(\frac{J_{\mathrm{SC}}}{J_{0}}\right)$$

where *k*_B_ is Boltzmann constant, *T* is the temperature in Kelvin, *q* is the elementary charge, *J*_0_ is the dark current density, and *J*_SC_ is the short circuit current density. Shunt resistance (*R*_SH_) and series resistance (*R*_S_) can be roughly evaluated by comparing the dark current. For TA‐free device, the lower leakage current under negative bias suggests a higher *R*_SH_ while higher current in high bias region shows a lower *R*_S_. Exact resistance data can be extracted from the *J–V* curves (Figure S15a, Supporting Information) and the *R*_S_ and *R*_SH_ are 3.29 Ω cm^2^ and 1625.59 Ω cm^2^ for TA‐free device while the values are 5.80 Ω cm^2^ and 371.09 Ω cm^2^, respectively for TA device, which is consistent with the dark current measurement. The higher *R*_SH_ and lower *R*_S_ contribute to a higher FF in TA‐free device. We then studied the *V*_OC_ performance as a function of light intensity (*P*_light_) to evaluate the recombination process. The *P*_light_ dependent *V*_OC_ performance can be used to analyse the trap‐assisted charge recombination according to the relation of *V*_OC_ and *P*_light_ (*V*_OC_∝(*nkT*/*q*)ln*P*_light_). As shown in Figure S15c, Supporting Information, in semi‐logarithmic coordinates, the slope of the fitting line for *V*_OC_ versus *P*_light_ gives the ideal factor (*n*) in *nk*_B_ *T*/*q*. The deviation of the slope from *k*_B_ *T*/*q* reflects the occurrence of trap‐induced recombination. The slope for TA‐free device was 2.11 *k*_B_ *T*/*q* whereas slope for the TA device was 2.14 *k*_B_ *T*/*q*. The slightly higher *n* value of TA device suggests higher ratio of trap‐assisted Shockley–Read–Hall (SRH) recombination in device, which is expected to be caused by the higher resistance of Ag/PFN‐Br interface after annealing. It is worth noting that the *n* value for tandem device is significantly higher than the value reported in single junction solar cells, which may be due to the recombination at tunnel junction (interconnecting layer) in tandem devices. Figure S16, Supporting Information, presents the electroluminescence (EL) spectrum of CsPbI_2_Br/PM6:Y6 tandem device under forward bias ranging from ≈0–4 V. Two EL peaks located at 645 nm (1.92 eV) and 906 nm (1.37 eV) can be ascribed to the radiative recombination in CsPbI_2_Br and PM6:Y6 BHJ film, respectively. Comparing with TA device, TA‐free device exhibits an excellent EL performance under a given current injection, suggesting higher carrier densities in sub‐cells and a higher radiative recombination rate. The higher EL also further implies the suppressed nonradiative recombination due to the lower defect density and charge transport resistance within the device, which benefits the charge collection process under illumination. Therefore, TA‐free device exhibits a lower *E*_loss_ induced by charge trapping and produces a higher voltage, which is consistent with the *E*_loss_ test in organic sub‐cell.

*J*–*V* curves measured under AM 1.5G illumination for the best performing individual sub‐cells and TA‐free perovskite/organic TSCs are presented in Figure 7b and the photovoltaic parameters are summarized in **Table** **1**. The best performing tandem device achieved a PCE of 20.6% under reverse scan with a *V*_OC_ of 2.097 V, *J*_SC_ of 13.09 mA cm^−2^, and FF of 75.1%, which is one of the highest PCEs reported based on perovskite/organic TSCs (Table S6, Supporting Information) so far. Figure 7c presents the EQE spectra of sub‐cells measured separately in tandem device by sequentially applying bias light with 550 nm short pass filter and 800 nm long pass filter. The EQE curves of the sub‐cells divide the incident light spectrum evenly into two parts, showing their complementary characteristics. The EQE spectrum of entire TSC is shown in dotted line, which was obtained by superimposing two EQE spectra. The integrated current densities *J*_EQE_s for the front cell and rear cell are 12.47 mA cm^−2^ and 12.86 mA cm^−2^, respectively, which are well‐matched. The *J*_EQE_ is also consistent with the *J*_SC_ obtained from the *J*–V curve with less than 5% difference. To investigate the photostability under constant AM 1.5G illumination, we performed the maximum power point (MPP) tracking, and the results are shown in Figure 7d. The tandem device maintained a stable current output under 1.74 V bias, showing a stable PCE of 19% with negligible degradation for 450 s. Figure 7e shows the statistic histogram of PCE values for 31 cells from the same batch; the histogram shows that the efficiency is normally distributed between 16.5% and 20.6%. Besides, the detailed distribution of *V*_OC_, *J*_SC_, and FF is depicted in Figure S17, Supporting Information, and the extracted data are shown in Table S5, Supporting Information. An extremely high average *V*_OC_ of 2.081 V was obtained; the maximum *V*_OC_ reached a record high of 2.116 V (Figure S18, Supporting Information) with a *J*_SC_ of 12.68 mA cm^−2^, FF of 75.2%, and PCE of 20.2%. It is worth noticing that the voltage of TSC is almost the sum of perovskite and organic sub‐cells, with a voltage loss of only ≈0.001 V. The magnificent *V*_OC_ indicates a well‐constructed ICL layer that minimizes the energy loss caused by nonradiative recombination happening in the ICL/sub‐cell interfaces, and thus, the high *V*_OC_ of sub‐cells can eventually contribute to final output. As shown in Movie S1, Supporting Information, our 2T‐TSC can light up a red LED due to high output voltage. Large area (1 cm^2^) device was also prepared and achieved a promising PCE of 16.5% with *V*_OC_ of 2.039 V, *J*_SC_ of 11.24 mA cm^−2^, and FF of 71.8% (Figure S19, Supporting Information), surpassing the large area single junction organic solar cell efficiency reported so far.^[^ ^36^ ^]^ We further tested the stability of tandem device because all–inorganic CsPbI_2_Br are reported to have multi‐phase transition under stimulation of moisture or illumination and face serious ions migration‐induced trap assisted recombination; the results are shown in Figure 7f.^[^ ^37^ ^]^ The device maintained a good stability in N_2_ glovebox for 700 h, with initial PCE value of 18.9% and the final PCE of 20.3%. It should be noted that the tandem device has shown light healing effect during stability test, as shown in Figure S20, Supporting Information. The device performance can be improved after illumination for several minutes;^[^ ^38^ ^]^ and hence, the photovoltaic parameters in stability tests were recorded after light soaking.

**Table 1:** Photovoltaic parameters of the optimal individual sub‐cells and TA‐free TSC

Device	*V*_OC_ [V]	*J*_SC_ [mA cm^−2^]	FF [%]	PCE [%]
CsPbI_2_Br	1.271	14.79	78.1	14.7
PM6:Y6	0.846	22.37	70.7	13.4
TA‐free 2T‐TSC	2.097	13.09	75.1	20.6

## 3 Conclusion

In summary, we have presented a high‐performance 2T all‐inorganic perovskite/organic tandem solar cell consisting of absorption well‐matched CsPbI_2_Br and PM6:Y6 blend as front and rear cell absorber, respectively. The inserting of s‐ZnO layer in perovskite sub‐cell facilitates the charge separation and enhances *V*_OC_. We found that thermal annealing eliminates a key charge transport process at the electrode/PFN‐Br interface in organic sub‐cell, leading to undesirable charge accumulation and recombination. The increased charge transport resistance at interface causes higher energy loss through nonradiative recombination while the TA‐free device obtained an improved *V*_OC_ without compromising the *J*_SC_ and FF. 2T‐TSC renders a remarkable PCE of 20.6% for small‐area cell and 16.5% for large‐area cell. The outstanding *V*_OC_ of 2.116 V is approximately the sum of V_OC_ of individual sub‐cells with only ≈0.001V difference. High efficiency and *V*_OC_ of our 2T‐TSCs demonstrates that tandem with all‐inorganic perovskite is a promising strategy to break the efficiency bottleneck of 20% for organic solar cells.

## 4 Experimental Section

### Materials and Experimental Details

PbI_2_(99.99%) and PbBr_2_ (99.9%) were purchased from TCI. PM6, Y6, and PFN‐Br were purchased from Derthon Optoelectronic Material Science Technology Co., Ltd. SnO_2_ (15% in H_2_O colloidal dispersion) was purchased from Alfa Aesar. Poly(triarylamine) (PTAA) was purchased from Xi'an Polymer Light Technology Corp. Zinc acetate dihydrate (Zn(CH_3_COO)_2_·2H_2_O, 99.995%) and chlorobenzene were purchased from Aladdin. CsI (99.999%), *N,N*‐Dimethylformamide (DMF), and dimethylsulfoxide (DMSO) were purchased from J & K. Ethanolamine, 2‐Methoxyethanol, and isopropanol were purchased from Sigma–Aldrich.

### Solutions Preparation

0.1 m ZnO sol–gel solution was prepared by dissolving 21.95 mg Zn(CH_3_COO)_2_·2H_2_O in 1 mL 2‐Methoxyethanol and adding 6 µL of ethanolamine; the solution was stirred for 24 h before use. ZnO NPs were synthesized as previously reported.^[^ ^39^ ^]^ For CsPbI_2_Br perovskite, 0.8 m precursor solution was obtained by dissolving 207.8 mg CsI, 184.4 mg PbI_2_, and146.8 mg PbBr_2_ in 1 mL DMSO and DMF mixed solution (volume ratio 1:4). The solution was then stirred overnight at room temperature in a glovebox and filtered with a 0.22 µm PTFE filter before use. For PM6:Y6 blend, 14 mg mL^−1^ solution was prepared by dissolving 6.4 mg PM6 and 7.6 mg Y6 (weight ratio 1:1.2) in 1 mL chloroform (with extra 0.5% CN as additive). The solution was stirred at room temperature for over 3 h before use. PFN‐Br was dissolved in methanol with concentration of 0.5 mg mL^−1^.

### Fabrication of Single Junction Perovskite Solar Cells

The ITO substrates were cleaned sequentially by ultrasonicating in detergent solution, deionized water, acetone, and ethanol for 10 min each, followed by UV‐ozone treatment for 20 min. s‐ZnO layer was formed by spin coating ZnO sol–gel solution at 3000 rpm for 30 s, followed by annealing at 170 °C for 1 h. In comparison, ZnO NPs were spin coated at 3000 rpm for 30 s and then dried at 60 °C for 5 min. SnO_2_ was spin coated on ZnO layer at 3000 rpm for 30s, followed by annealing at 150 °C for 30 min. The CsPbI_2_Br precursor was spin coated on ZnO/SnO_2_ bilayer ETL in N_2_ filled glovebox via a two‐step spin‐coating program and followed a gradient annealing process; the depositing procedure was set to be 1000 rpm for 10 s and 3500 rpm for 25 s. 120 µL chlorobenzene was dripped onto the rotating substrate 10 s prior to the end of the program. The samples were immediately transferred to a hotplate and sequentially annealed at 50 °C for 1 min and 240 °C for 1 min. PTAA with concentration of 10 mg mL^−1^ was spin coated on perovskite at 3000 rpm for 30 s. Finally, MoO_3_ and Ag electrode were thermally evaporated with controlled thickness of 10 and 100 nm, respectively.

### Fabrication of Single Junction Organic Solar Cells

For PEDOT:PSS based device, PEDOT:PSS was spin coated on UV‐ozone treated ITO substrate at 3000 rpm for 40 s, and then annealed at 100 °C for 10 min. For PFN‐Br based device, PFN‐Br was deposited on ITO substrate at 3000 rpm for 30 s. The substrates were then transferred into an N_2_ filled glovebox. The PM6:Y6 (1:1.2) blend film was deposited by using dynamic spin coating at 2000 rpm for 30 s, followed by annealing at 110 °C for 10 min in the case of TA device. Then, MoO_3_ and Ag electrode were thermally evaporated with controlled thickness of 10 and 100 nm, respectively.

### Fabrication of Tandem Solar Cells

The ICL of MoO_3_ (≈10 nm)/Ag(≈1 nm)/PFN‐Br layer was formed by evaporating MoO_3_ and Ag; the PFN‐Br layer was spin coated at 3000 rpm for 30 s. Other procedures can be referred to the fabrication in single junction solar cell.

### Characterization

*J–V* measurement was conducted under AM 1.5 G illumination using a sunlight simulator (Enlitech, Sirius‐SS150A‐D) with a Keitheley 2400 source meter. EQE measurement was carried out with a QE‐R quantum efficiency system (Enlitech). For the tandem device, the EQE for sub‐cells was recorded applying bias light with 550 nm shortpass filter and 800 nm longpass filter, respectively. UV–vis spectra were measured using a PerkinElmer LAMBDA 950 spectrophotometer; samples were encapsulated in N_2_ atmosphere to avoid potential decomposition. Surface and cross‐section morphologies of perovskites and device were characterized using a Zeiss Gemini 300 SEM. AFM images were obtained by using a NanoMan VS microscope. GIWAXS was performed at the small‐angle X‐ray scattering (SAXS)/WAXS beamline at the Australian Synchrotron. The steady‐state PL and time‐resolved PL (TRPL) were measured using a FLS1000 photoluminescence spectrometer (Edinburgh Instruments). The samples were encapsulated and excited with 480 nm monochromatic light (for PL) and 510 nm pulsed laser (for TRPL). Transient photovoltage measurements, capacitance–voltage, and electrochemical impendence spectrum were carried out using a Paios instrument (FLUXiM AG). FTPS‐EQE was measured using an integrated system with Fourier transform photocurrentmeter (PECT‐600, Enlitech). EQEEL measurement was performed by high‐sensitivity solar cell electroluminscence (EL) efficiency measurement system (REPS, Enlitech).

### Statistical Analysis

Statistical analysis and data plotting were performed using OriginLab software. For easy comparison, transient photocurrent decay (Figure 1b), TRPL (Figure 1d), absorption spectra (Figures S4 and S13, Supporting Information), and XPS spectra (Figure S11, Supporting Information) were normalized. Average *V*_OC_ and the statistical distribution histograms in Figure S17, Supporting Information, and Figure 7e were obtained from 31 cells. The EIS parameters were extracted by fitting the curves using Zview. The scale bars of AFM and SEM images were presented in the corresponding figure. The data for the rest of the charts were directly obtained during the measurements.

## Conflict of Interest

The authors declare no conflict of interest.

## Author Contributions

X.G. and X.L. contributed equally to this work. *Conceptualization, methodology, and writing original draft*: X.G. *Measurement and data analysis*: X.L., Y.Z., T.W., W.T., Q.L., W.L, and C.S. Resources and data analysis: C.R.M. *Resources, review, and validating*: P.S. and F.H. *Conceptualization, project administration, funding acquisition, and writing: reviewing and editing*: A.K.K.K.

## Supporting information

Supporting Information

Supporting Video

## Data Availability

The data that support the findings of this study are available in the Supporting Information of this article.
