# Supplementary material for: Organic Solar Cell With Efficiency Over 20% and V OC Exceeding 2.1 V Enabled by Tandem With All‐Inorganic Perovskite and Thermal Annealing‐Free Process
Source: Adv Sci (Weinh). 2022 Jul 20;9(28):2200445. doi: 10.1002/advs.202200445 (PMC9534952; doi:10.1002/advs.202200445)
Supplement: Supplementary file 1 — Supporting Information [file ADVS-9-2200445-s002.pdf]

ADVANCED SCIENCE Open Access

Supporting Information

for *Adv. Sci.*, DOI 10.1002/advs.202200445

Organic Solar Cell With Efficiency Over 20% and V_{OC} Exceeding 2.1 V Enabled by Tandem With All-Inorganic Perovskite and Thermal Annealing-Free Process

*Xiaoyu Gu, Xue Lai, Yuniu Zhang, Teng Wang, Wen Liang Tan, Christopher R. McNeill, Qian Liu, Prashant Sonar, Feng He, Wenhui Li, Chengwei Shan and Aung Ko Ko Kyaw**

Supporting Information

Organic Solar Cell with Efficiency over 20% and V_{OC} Exceeding 2.1 V Enabled by Tandem with All-Inorganic Perovskite and Thermal Annealing-Free Process

*Xiaoyu Gu, Xue Lai, Yuniu Zhang, Teng Wang, Wen Liang Tan, Christopher R. McNeill, Qian Liu, Prashant Sonar, Feng He, Wenhui Li, Chengwei Shan, Aung Ko Ko Kyaw**

X. Gu, X. Lai, Y. Zhang, T. Wang, Q. Liu, W. Li, C. Shan, Prof. A. K. K. Kyaw
Guangdong University Key Laboratory for Advanced Quantum Dot Displays and Lighting, and Department of Electrical & Electronic Engineering, Southern University of Science and Technology, Shenzhen 518055, PR China
E-mail: aung@sustech.edu.cn

X. Lai, Prof. F. He
Department of Chemistry, Southern University of Science and Technology, Shenzhen 518055, PR China

W. Tan, Prof. C. R. McNeill
Department of Materials Science and Engineering, Monash University, Clayton, VIC 3800, Australia.

Q. Liu, Prof. P. Sonar
Center for Materials Science, Queensland University of Technology, Brisbane, QLD 4000, Australia.

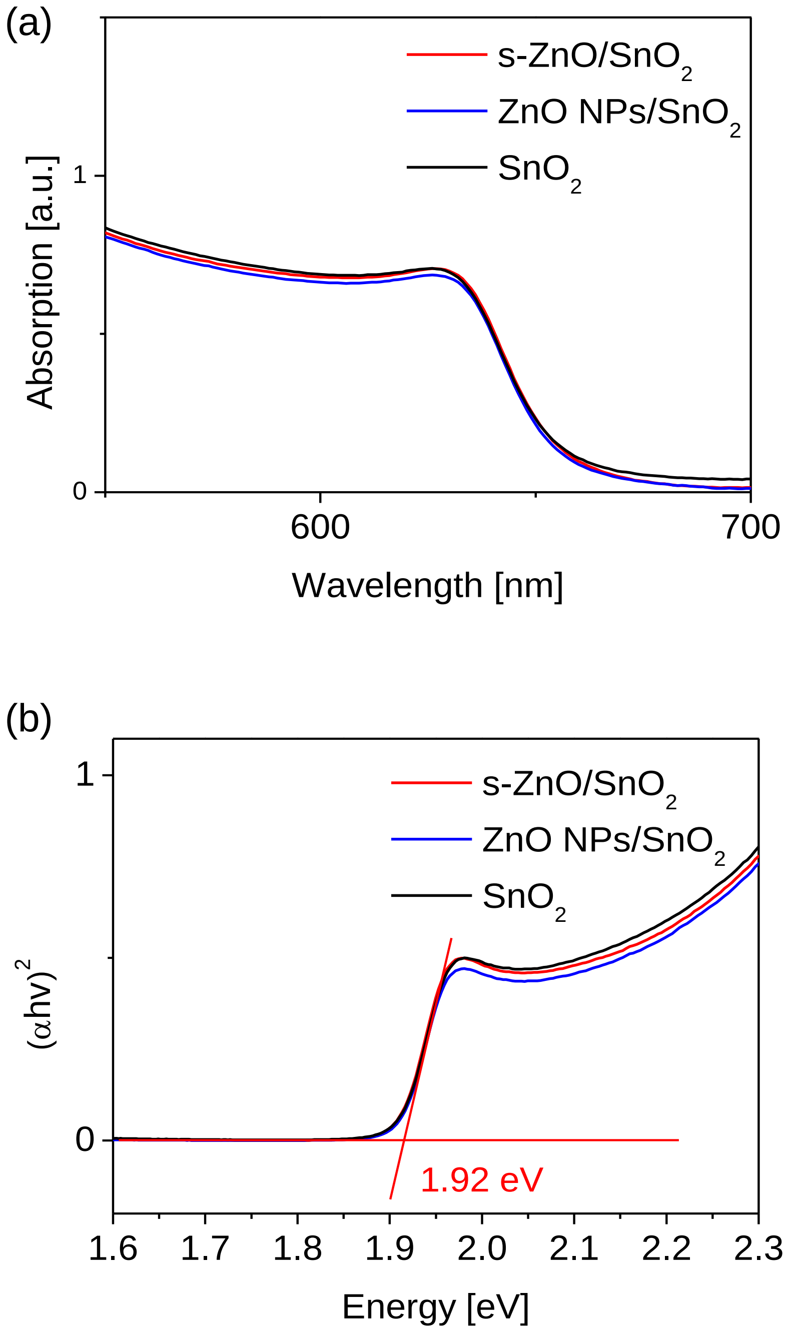

Figure S1. (a) UV-vis absorption spectrum of $CsPbI_2Br$ films deposited on SnO_2, ZnO NPs/SnO_2 and s-ZnO/SnO_2 and (b) the corresponding Tauc plots.

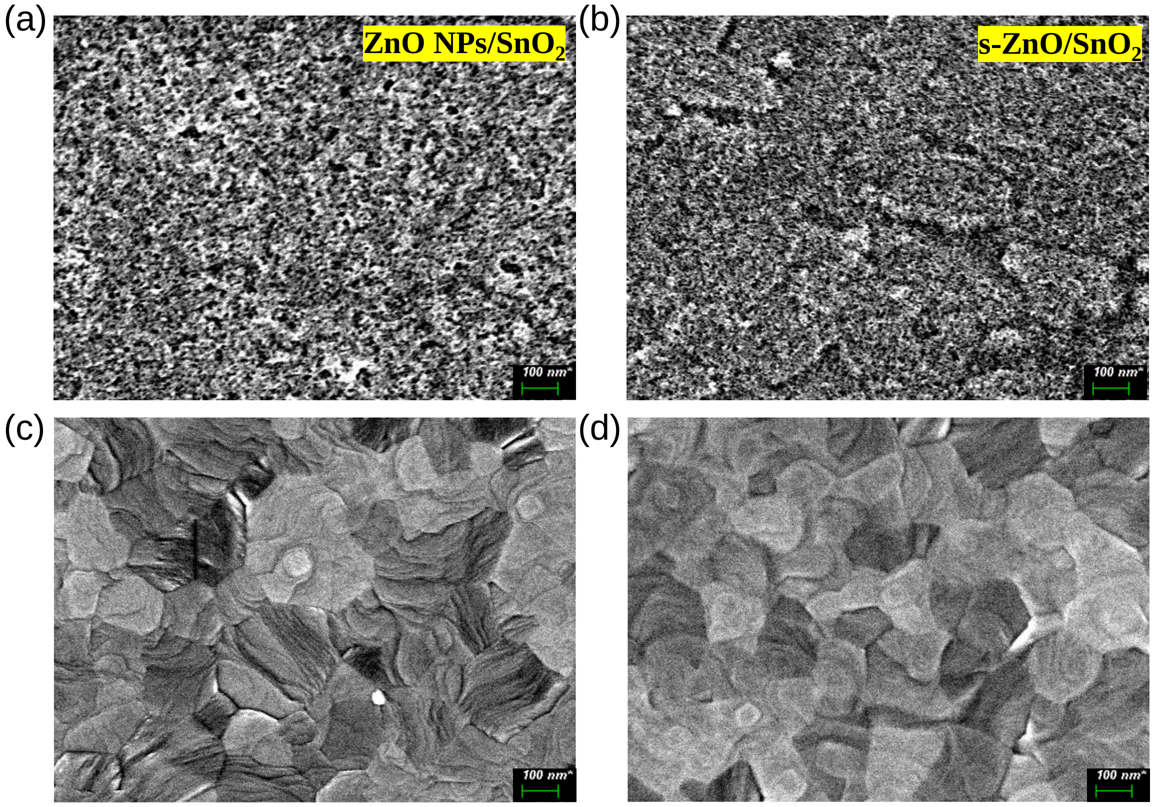

Figure S2. Surface SEM images of (a) ZnO NPs/SnO_2, (b) s-ZnO/SnO_2, (c) $CsPbI_2Br$ on ZnO NPs/SnO_2, (d) $CsPbI_2Br$ on s-ZnO/SnO_2.

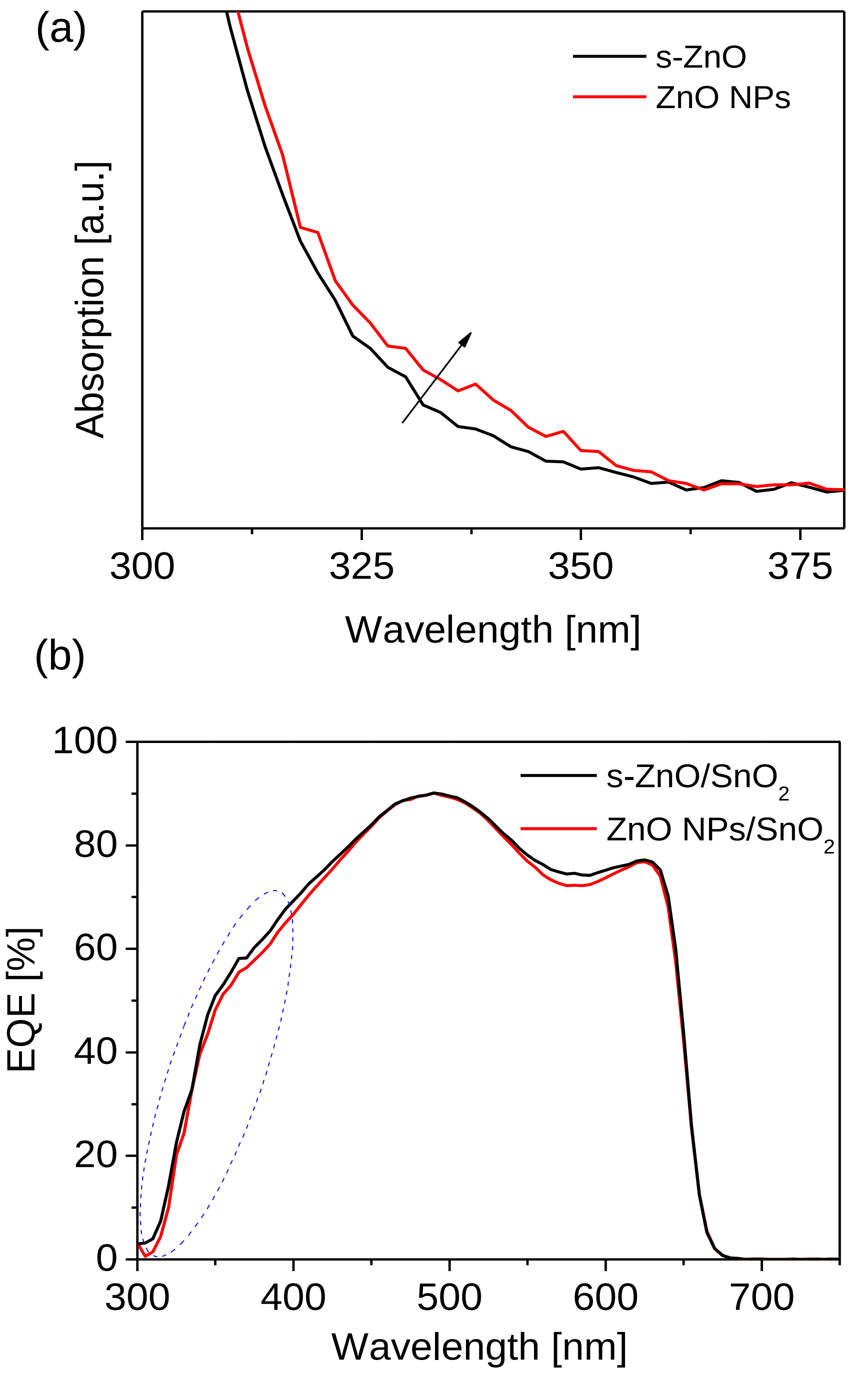

Figure S3. (a) UV-Vis absorption spectra of s-ZnO and ZnO NPs films. (b) EQE spectra of all-inorganic perovskite front sub-cells based on s-ZnO/SnO_2 and ZnO NPs/SnO_2 ETLs.

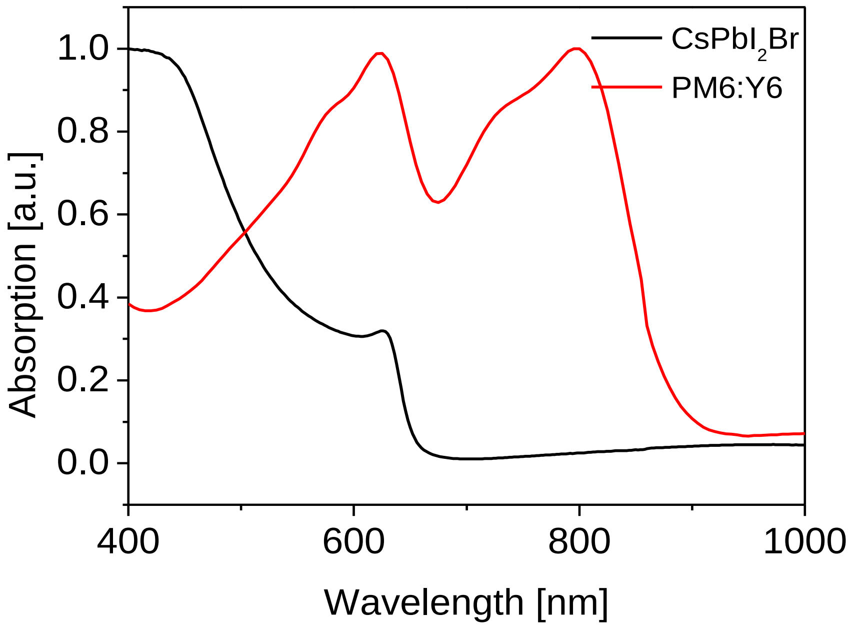

Figure S4. Absorption spectrum of $CsPbI_2Br$ film and PM6:Y6 blends.

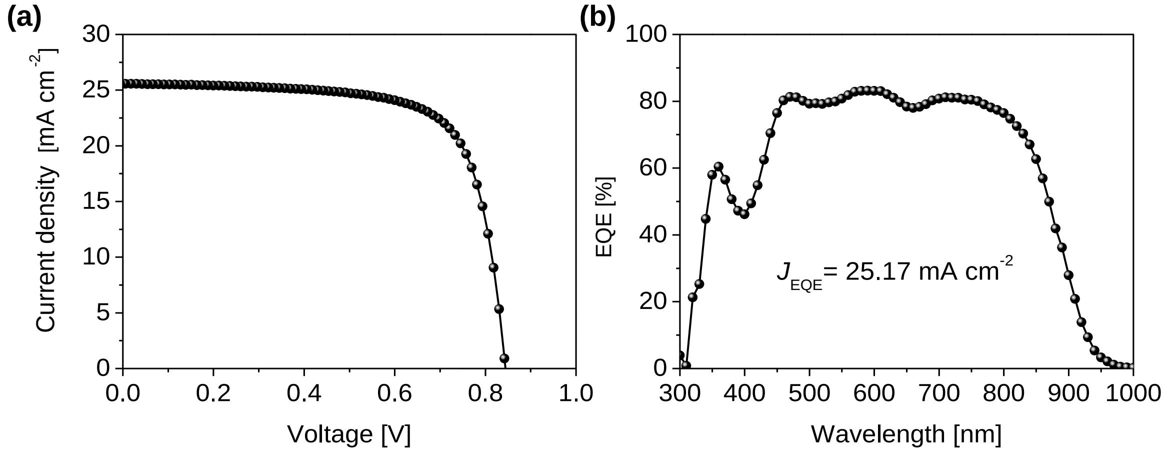

Figure S5. (a) *J-V* curves and (b) EQE curves of single junction organic solar cell with standard structure of ITO/PEDOT:PSS/PM6:Y6/MoO_3/Ag.

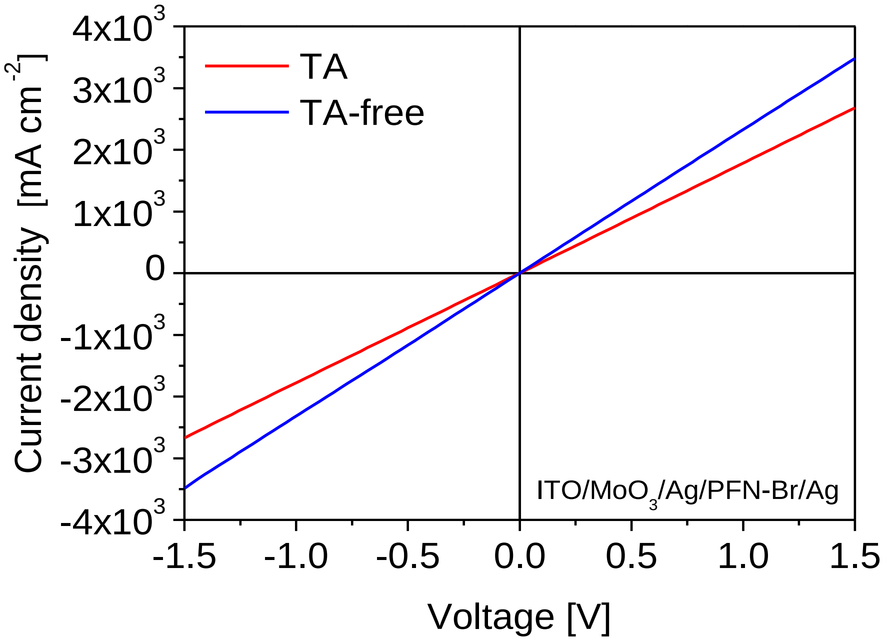

Figure S6. *J-V* curves of TA and TA-free ICL.

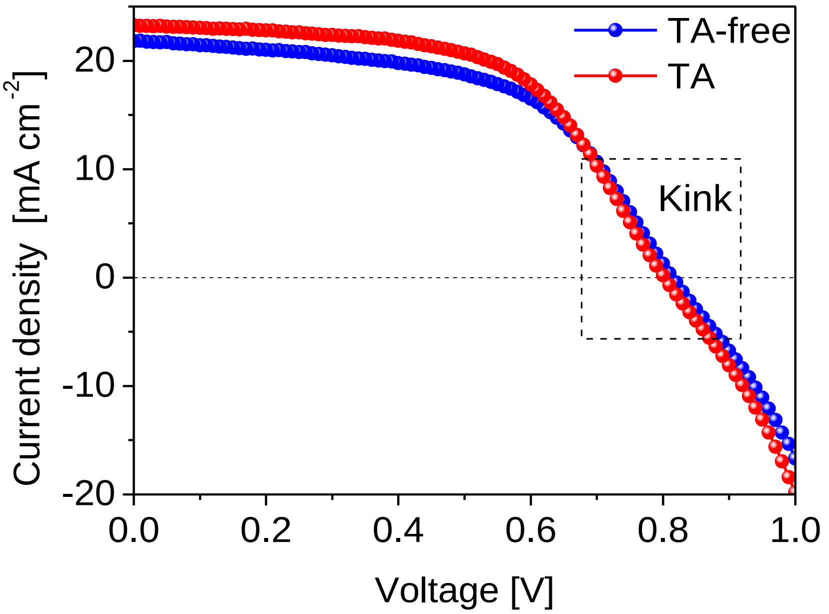

Figure S7. J-V curves for TA and TA-free organic rear sub-cell with structure of ITO/PFN-Br/PM6:Y6/MoO_3/Ag.

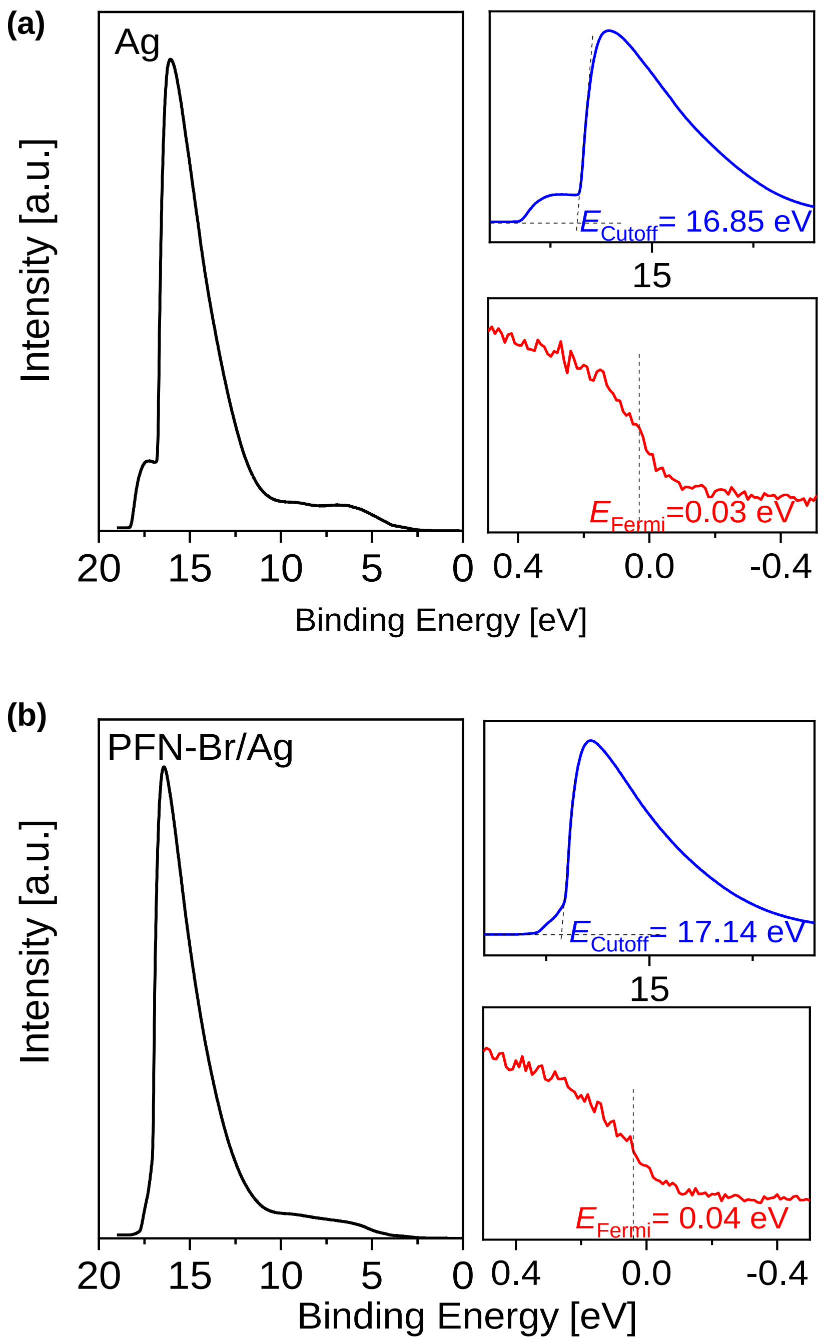

Figure S8. UPS spectra of (a) ITO/Ag and (b) ITO/PFN-Br/Ag

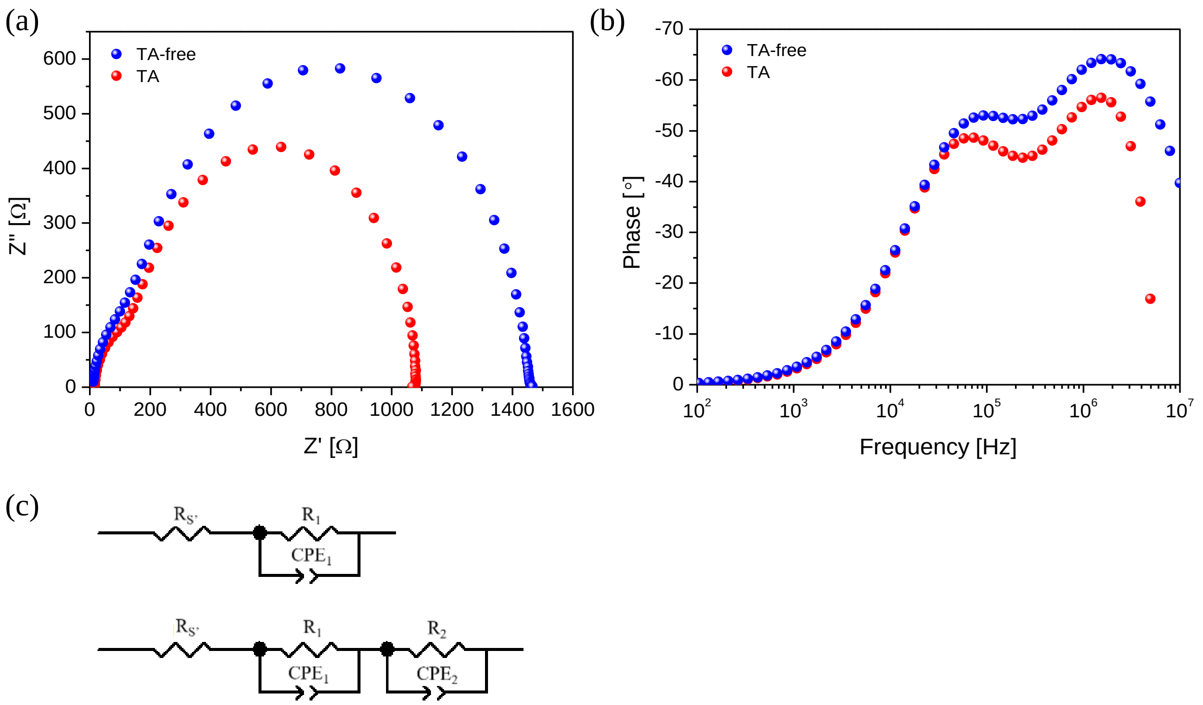

Figure S9. (a) Nyquist plot and (b) Bode plot of TA and TA-free organic rear sub-cells based on ITO/PFN-Br substrates. (c) Equivalent circuits used to fit spectra in Figure 4.

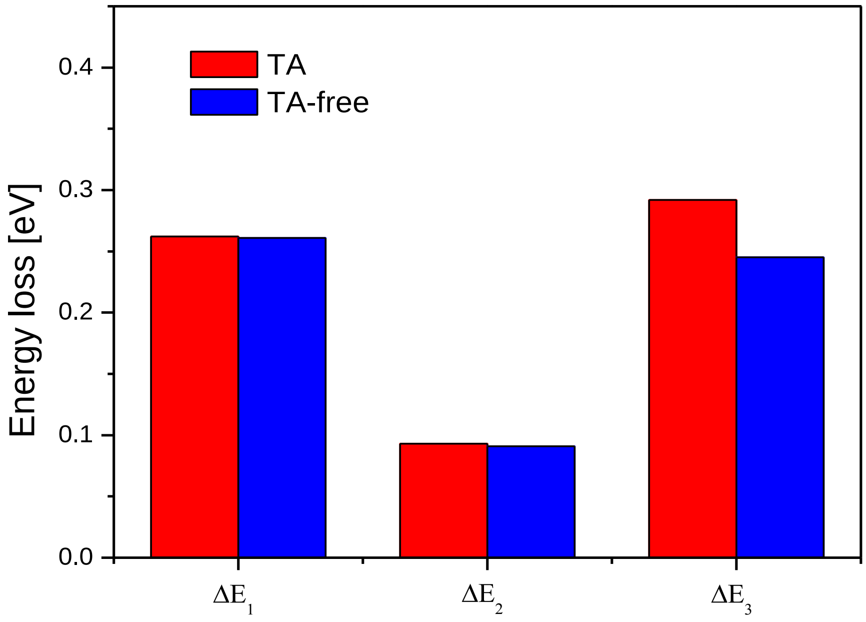

Figure S10. Energy losses for TA and TA-free organic rear sub-cells.

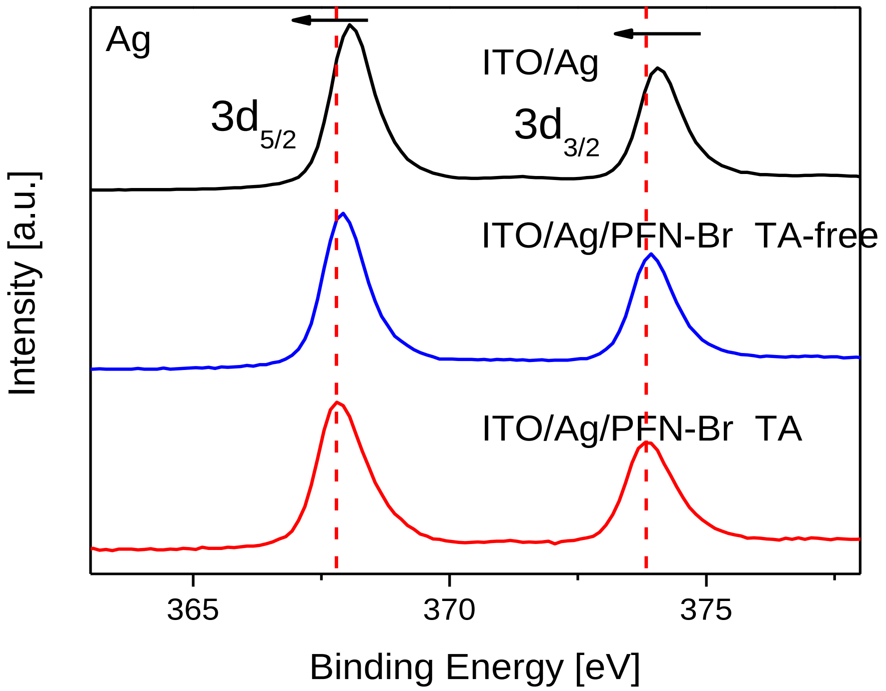

Figure S11. XPS spectra of Ag 3d regions for different substrates.

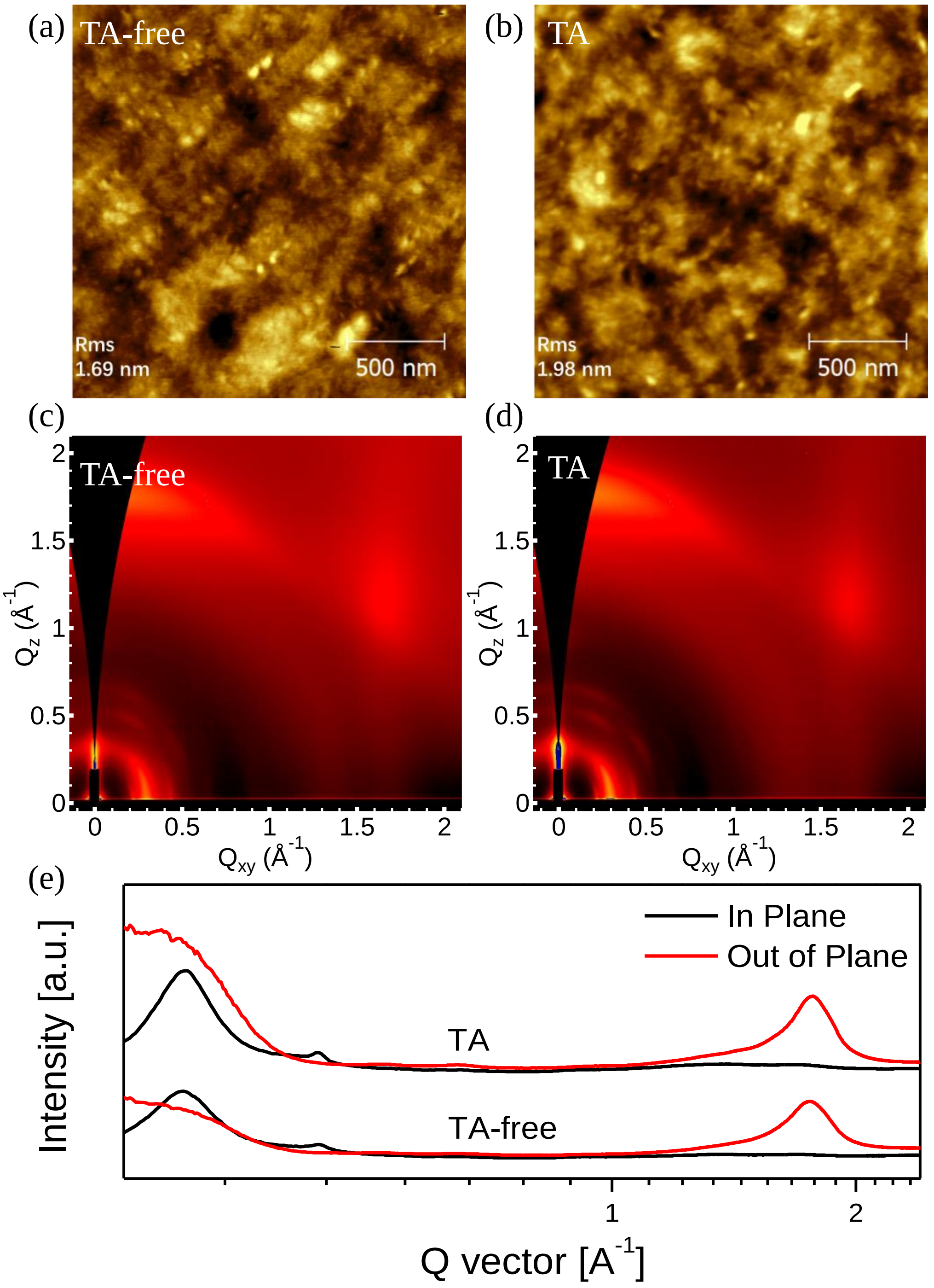

Figure S12. (a-b) AFM images of the as-cast and thermal annealed PM6:Y6 blend films; (c-d) GIWAXS images for as cast and thermal annealed PM6:Y6 blend films; (e) GIWAXS scattering intensity along the in-plane and out-of-plane directions.

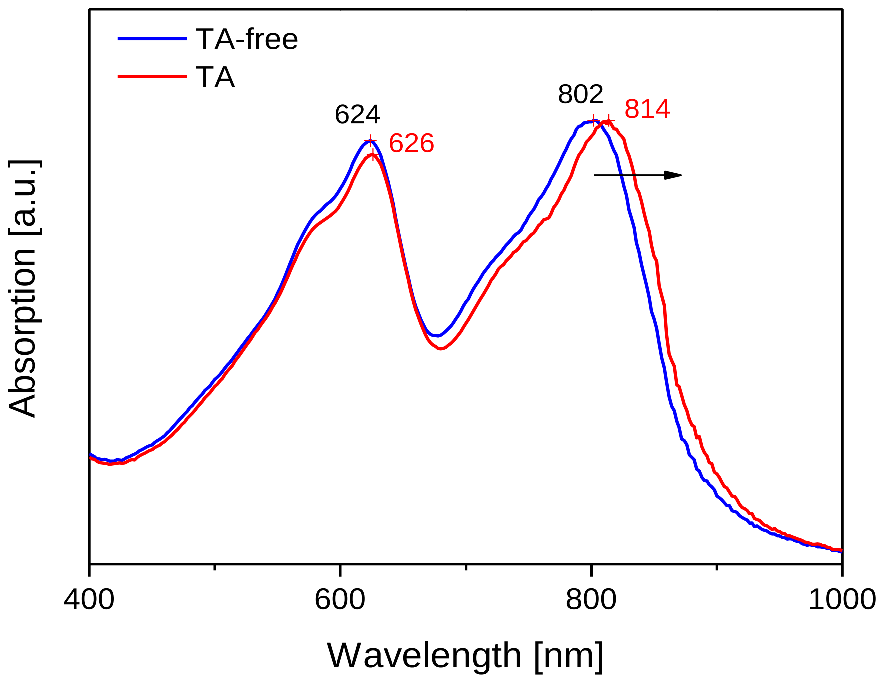

Figure S13. Absorption spectra for 110°C TA film and TA-free film.

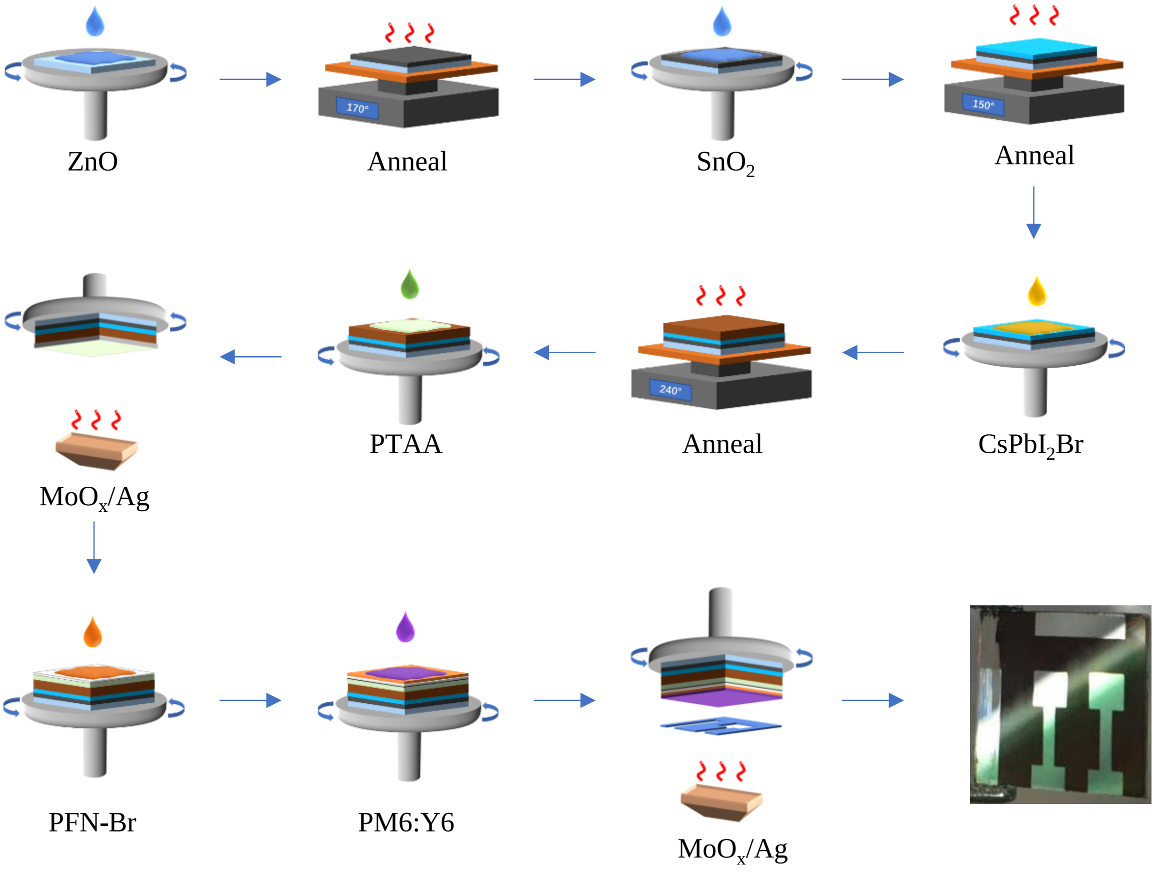

Figure S14. Fabrication process for 2T- inorganic perovskite/organic TSCs.

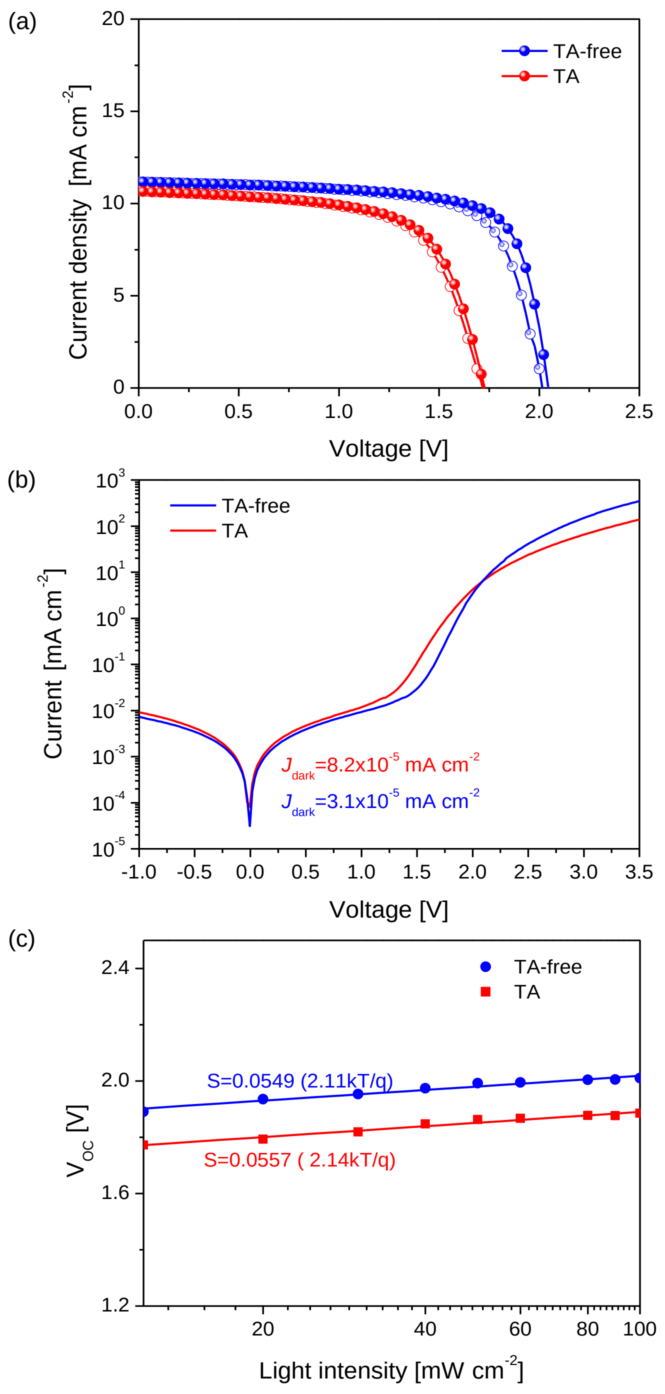

Figure S15. (a) *J-V* curves, (b) dark current and (c) light intensity dependent V_{OC} curves for TA and TA-free TSCs.

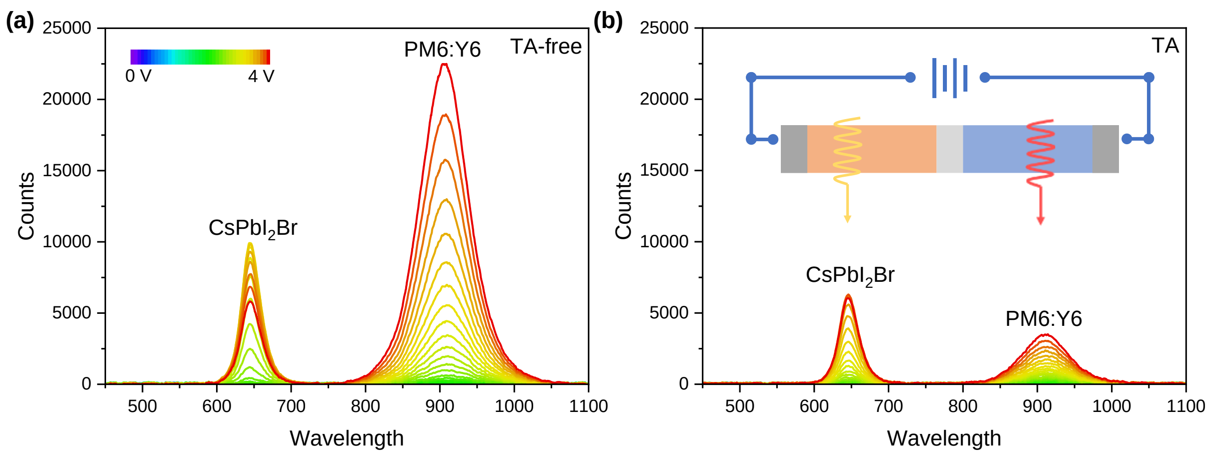

Figure S16. Electroluminescence spectra of CsPbI2Br/PM6:Y6 tandem solar cell under different bias voltage for (a) TA-free device and (b) TA device

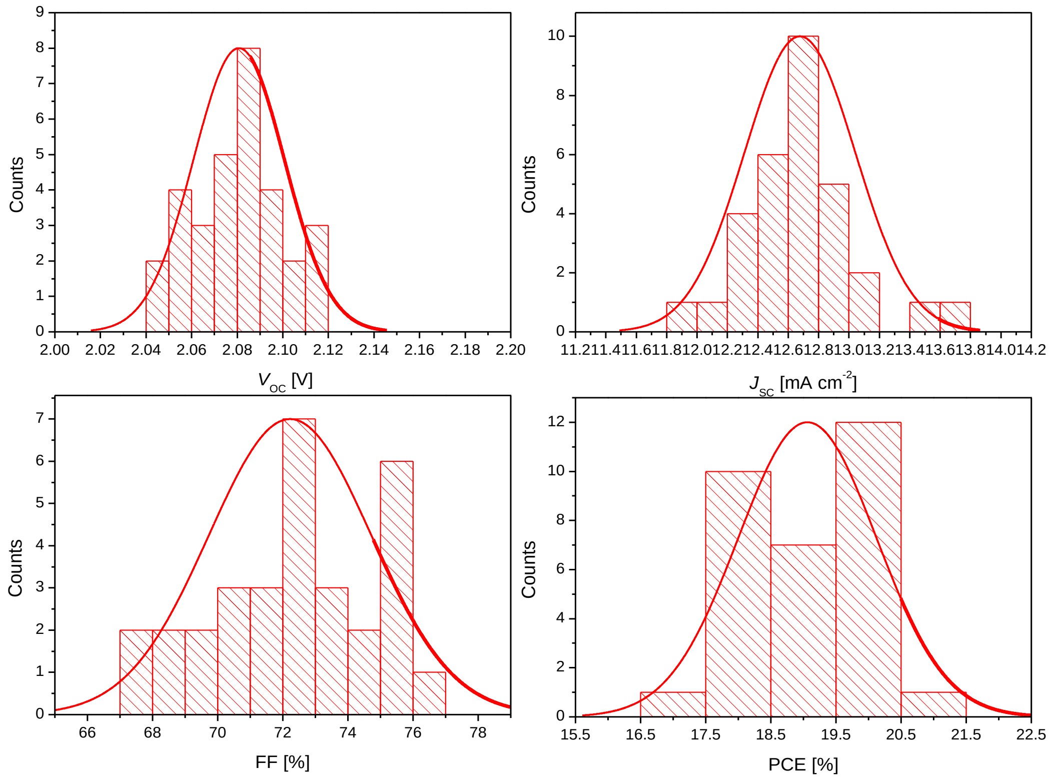

Figure S17. Distribution histograms of V_{OC}, J_{SC}, FF and PCE for 31 cells from the same batch.

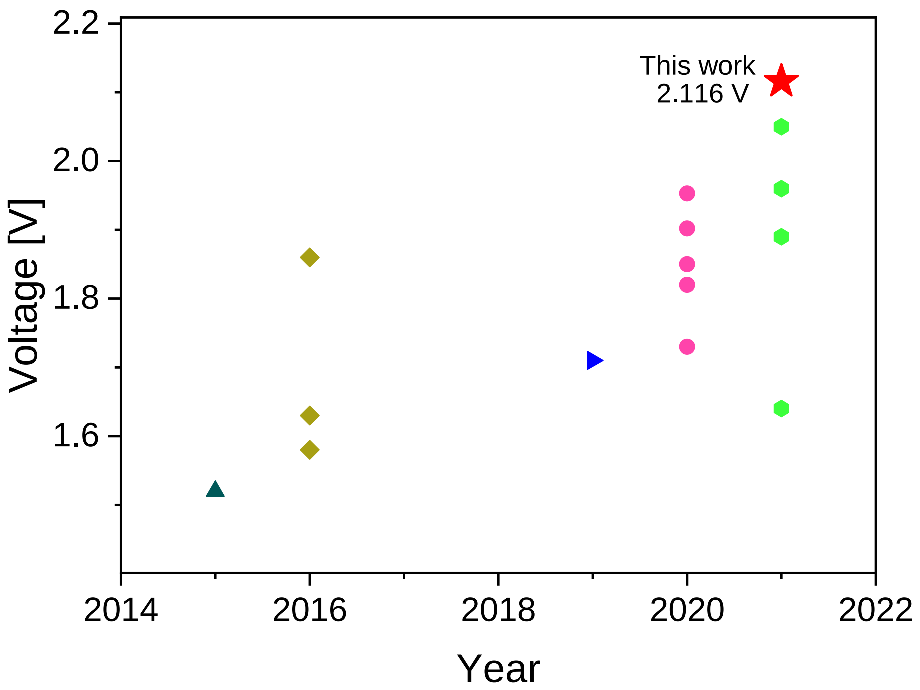

Figure S18. Statistic summary of V_{OC} for perovskite/organic 2T-TSCs in recently years.

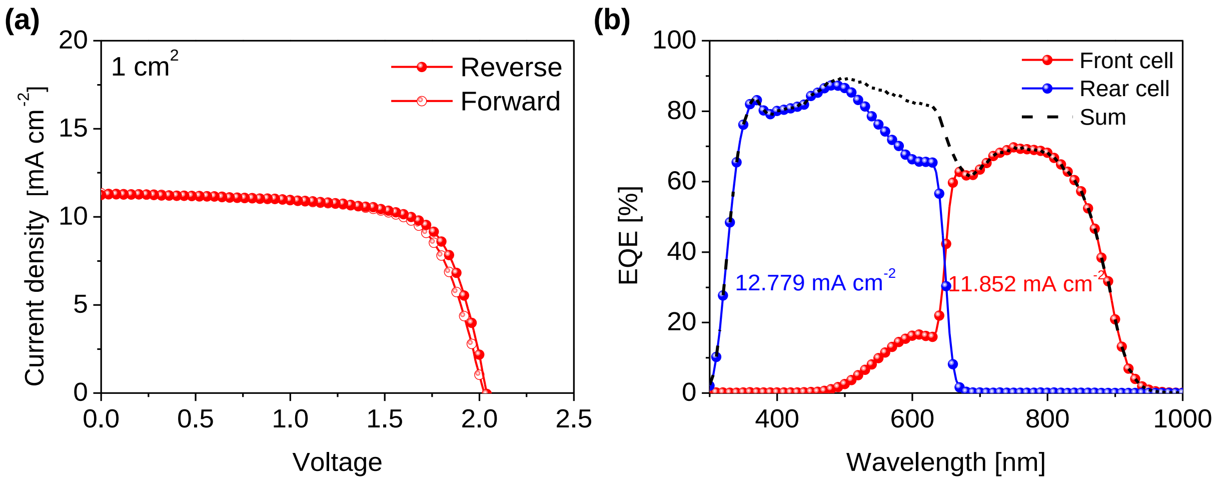

Figure S19. (a) *J-V* curves of TA-free TSC with area of 1 cm^2. (b) EQE spectra of the corresponding TSC.

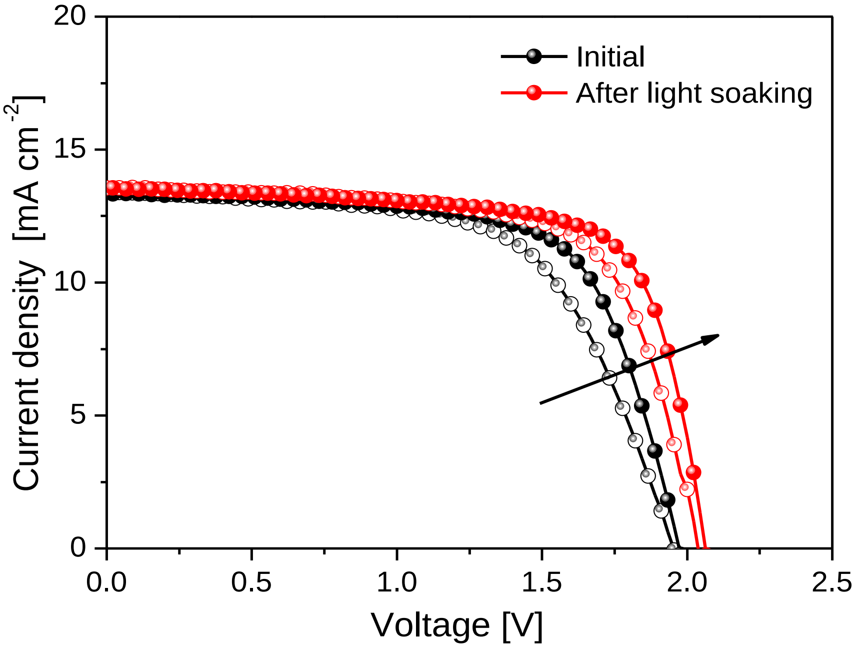

Figure S20. Device performance variation after light soaking.

Table S1. Photovoltaic parameters of all-inorganic perovskite front sub-cells based on different ETLs

ETL	V_{OC} [V]	J_{SC} [mA cm^{-2}]	FF [%]	PCE [%]
SnO_2	1.097	14.00	77.8	12.0
ZnO NPs/SnO_2	1.177	13.75	76.0	12.3
s-ZnO/SnO_2	1.271	14.79	78.1	14.7

Table S2. Photovoltaic parameters of TA and TA-free organic rear sub-cells fabricated on ITO/Ag/PFN-Br substrates.

Device	V_{OC} [V]	J_{SC} [mA cm^{-2}]	J_{EQE} [mA cm^{-2}]	FF [%]	PCE [%]	R_S [Ω]	R_{SH} [Ω]
TA	0.665	21.04	20.02	51.7	7.2	143.3	3826.4
TA-free	0.846	22.37	22.14	70.7	13.4	86.6	13949.5

Table S3. Fitting parameters of EIS for TA and TA-free organic rear sub-cell devices.

Device	R_1 [Ω]	R_2 [Ω]	R_3 [Ω]	CPE_{T1} [F]	$CPEP_1$	CPE_{T2} [F]	CPE_{P2}
TA	9.231	135.5		3.984×10^{-9}	0.958		
TA-free	8.516	68.9	244.9	8.107×10^{-9}	0.924	1.570×10^{-8}	0.981

Table S4. Fitting parameters of energy losses in TA and TA-free organic rear sub-cell.

Device	E_g [eV]	ΔE_1 [eV]	ΔE_2 [eV]	ΔE_3 [eV]
TA	1.47	0.262	0.093	0.292
TA-free	1.49	0.261	0.091	0.245

Table S5. Photovoltaic parameters for 31 tandem cells from the same batch.

No.	V_{OC} [V]	J_{SC} [mA cm^{-2}]	FF [%]	PCE [%]
1	2.116	12.68	75.2	20.2
2	2.079	12.22	72.5	18.4
3	2.081	12.5	70.1	18.2
4	2.092	12.5	70.2	18.3
5	2.101	12.78	72.5	19.5
6	2.043	12.5	69.4	17.8
7	2.048	11.88	67.7	16.5
8	2.072	12.35	68.5	17.5
9	2.075	12.53	67.7	17.6
10	2.116	12.84	73.7	20.0
11	2.058	12.75	69.5	18.2
12	2.056	12.85	68.5	18.1
13	2.089	12.88	71.3	19.2
14	2.057	12.74	73	19.1
15	2.095	12.73	72.6	19.4
16	2.051	12.17	72	18.0
17	2.115	12.95	74.1	20.3
18	2.086	12.6	71	18.7
19	2.086	13.55	72	20.3
20	2.085	13.63	70.5	20.0
21	2.105	12.69	74.2	19.8
22	2.061	12.67	73	19.1
23	2.096	12.52	72.4	19.0

24	2.089	12.38	76.4	19.8
25	2.079	12.72	75.9	20.1
26	2.086	12.67	75	19.8
27	2.078	12.4	72.3	18.6
28	2.097	13.09	75.1	20.6
29	2.061	12.22	71.6	18.0
30	2.065	13.1	75.4	20.4
31	2.085	12.9	75.6	20.4

Table S6. Summary of parameters for monolithic perovskite/organic TSCs reported so far.

Year	Perovskite absorber	Eg eV	Organic absorber	V_{OC} V	V_{Loss} V	J_{SC} mA cm^{-2}	FF %	PCE %	Ref
2022	$CsPbI_2Br$	1.92	PM6:Y6	2.097 (2.116)	0.001	13.09	75.1	20.6	This work
2021	$CsPbI_{2.1}Br_{0.9}$	1.79	PM6:Y6	1.89	0.09	12.77	74.81	18.06	[1]
2021	$CsPbI_{1.8}Br_{1.2}$	1.93	PM6:Y6	2.05	0.03	13.36	76.82	21.04	[2]
2021	$CsPbI_2Br$		PM6:Y6-BO	1.96	0.06	13.3	80.8	21.1	[3]
2021	$CsPbI_2Br$	1.92	D18:Y6	2.05	0.03	13.07	75.3	20.18	[4]
2020	$MAPbI_3$	1.55	PTB7-DT:PC_{71}BM:O6t-4F	1.64	0.16	15.32	68.3	17.16	[5]
2020	$CsPbI_2Br$	1.9	PM6:Y6	1.953	0.097	12.46	75.59	18.38	[6]
2020	$CsPbI_2Br$	1.92	PTB7-Th:IEICO-4F	1.82	0.11	13.20	71.68	17.24	[7]
2020	$CsPbI_2Br$	1.85	PTB7-Th:IEICO-4F	1.73	0.12	12.94	81.0	18.04	[8]
2020	$Cs_{0.1}(FA_{0.6}MA_{0.4})_{0.9}Pb(I_{0.6}Br_{0.4})_3$	1.74	PBDB-T:SN6IC-4F	1.85	0.14	11.52	70.98	15.13	[9]
2020	$FA_{0.8}MA_{0.02}Cs_{0.18}PbI_{1.8}Br_{1.2}$	1.77	PBDBT-2F:Y6:PC_{71}BM	1.902	0.065	13.05	83.1	20.6	[10]
2019	$CsPbI_2Br$	1.92	PTB7-Th:COi8DFIC:PC_{71}BM	1.71	0.16	11.98	73.4	15.04	[11]
2016	$CH_3NH_3PbI_{3-x}Cl_x$		PBDTT-DPP:PC_{70}BM	1.58	0.02	8.02	68.0	8.62	[12]
2016	$MAPbI_3$	1.5	PCE-10:PC_{71}BM	1.63	0	13.1	75.1	16.0	[13]
2016	$CH_3NH_3PbI_3$		PSEHTT: IC_{60}BA	1.86	0.12	8.73	72.0	11.28	[14]
2015	$CH_3NH_3PbI_3$		PBSeDTEG8:PCBM	1.52	0.07	10.05	67.0	10.23	[15]

References

[1] X. Wu, Y. Z. Liu, F. Qi, F. Lin, H. T. Fu, K. Jiang, S. F. Wu, L. Y. Bi, D. Wang, F. Xu, A. K. Y. Jen, Z. L. Zhu, *J. Mater. Chem. A* **2021**, *9*, 10.
[2] W. Chen, D. Li, X. Chen, H. Chen, S. Liu, H. Yang, X. Li, Y. Shen, X. Ou, Y. Yang, L. Jiang, Y. Li, Y. Li, *Adv. Funct. Mater.* **2021**, 2109321.
[3] P. Wang, W. Li, O. J. Sandberg, C. Guo, R. Sun, H. Wang, D. Li, H. Zhang, S. Cheng, D. Liu, J. Min, A. Armin, T. Wang, *Nano Lett.* **2021**, *21*, 7845.
[4] L. Liu, Z. Xiao, C. Zuo, L. Ding, *J. Semicond.* **2021**, *42*, 020501.
[5] T. Zhu, Y. Yang, Y. Liu, R. Lopez-Hallman, Z. Ma, L. Liu, X. Gong, *Nano Energy* **2020**, *78*, 105397.
[6] S. Xie, R. Xia, Z. Chen, J. Tian, L. Yan, M. Ren, Z. Li, G. Zhang, Q. Xue, H.-L. Yip, Y. Cao, *Nano Energy* **2020**, *78*, 105238.
[7] K. Lang, Q. Guo, Z. He, Y. Bai, J. Yao, M. Wakeel, M. S. Alhodaly, T. Hayat, Z. Tan, *J. Phys. Chem. Lett.* **2020**, *11*, 9596.
[8] H. Aqoma, I. F. Imran, F. T. A. Wibowo, N. V. Krishna, W. Lee, A. K. Sarker, D. Y. Ryu, S. Y. Jang, *Adv. Energy Mater.* **2020**, *10*, 2001188.
[9] Z. Li, S. Wu, J. Zhang, K. C. Lee, H. Lei, F. Lin, Z. Wang, Z. Zhu, A. K. Y. Jen, *Adv. Energy Mater.* **2020**, *10*, 2000361.
[10] X. Chen, Z. Jia, Z. Chen, T. Jiang, L. Bai, F. Tao, J. Chen, X. Chen, T. Liu, X. Xu, C. Yang, W. Shen, W. E. I. Sha, H. Zhu, Y. Yang, *Joule* **2020**, *4*, 1594.
[11] Q. Zeng, L. Liu, Z. Xiao, F. Liu, Y. Hua, Y. Yuan, L. Ding, *Sci. Bull.* **2019**, *64*, 885.
[12] J. Liu, S. Lu, L. Zhu, X. Li, W. C. Choy, *Nanoscale* **2016**, *8*, 3638.
[13] Y. Liu, L. A. Renna, M. Bag, Z. A. Page, P. Kim, J. Choi, T. Emrick, D. Venkataraman, T. P. Russell, *ACS Appl. Mater. Interfaces* **2016**, *8*, 7070.
[14] A. R. Bin, M. Yusoff, J. Jang, *Chem. Commun.* **2016**, *52*, 5824.
[15] C.-C. Chen, S.-H. Bae, W.-H. Chang, Z. Hong, G. Li, Q. Chen, H. Zhou, Y. Yang, *Mater. Horiz.* **2015**, *2*, 203.
